# The Relative Content and Distribution of Absorbed Volatile Organic Compounds in Rats Administered Asari Radix et Rhizoma Are Different between Powder- and Decoction-Treated Groups

**DOI:** 10.3390/molecules25194441

**Published:** 2020-09-27

**Authors:** Guang-Xue Liu, Feng Xu, Ming-Ying Shang, Xuan Wang, Shao-Qing Cai

**Affiliations:** 1 Division of Pharmacognosy, School of Pharmaceutical Sciences, Peking University Health Science Center, Beijing 100191, China; guangxl@bjmu.edu.cn (G.-X.L.); xufeng_pharm@163.com (F.X.); 2 Department of Chemical Biology, School of Pharmaceutical Sciences, Peking University Health Science Center, Beijing 100191, China; xuanwang6818@bjmu.edu.cn

**Keywords:** Asari Radix et Rhizoma, volatile compounds, distribution, HS–SPME–GC–MS, metabolite profile

## Abstract

Asari Radix et Rhizoma (ARR) is an important traditional Chinese medicine. Volatile organic compounds (VOCs) are the main active constituents of ARR. Research on the metabolite profile of VOCs and the difference of absorbed constituents in vivo after an administration of ARR decoction and powder will be helpful to understand the pharmacological activity and safety of ARR. In this study, headspace solid-phase microextraction gas chromatography mass spectrometry (HS–SPME–GC–MS) was applied to profile the VOCs from ARR in rats in vivo. A total of 153 VOCs were tentatively identified; 101 were original constituents of ARR (98 in the powder-treated group and 43 in the decoction-treated group) and 15 were metabolites, and their metabolic reactions were mainly oxidation and reduction, with only two cases of methylation and esterification, and 37 unclassified compounds were identified only in the ARR-treated group. Of the 153 VOCs identified, 131 were reported in rats after oral administration of ARR for the first time, containing 79 original constituents, 15 metabolites, and 37 unclassified compounds. In the powder-treated group, methyleugenol, safrole, 3,5-dimethoxytoluene (3,5-DMT), 2,3,5-trimethoxytoluene (2,3,5-TMT), and 3,4,5-trimethoxytoluene (3,4,5-TMT) were the main absorbed constituents, the relative contents of which were significantly higher compared to the decoction-treated group, especially methyleugenol, safrole, and 3,5-DMT. In the decoction-treated group, 3,4,5-TMT, 2,3,5-TMT, kakuol, and eugenol were the main constituents with a higher content and wider distribution. The results of this study provide a reference for evaluating the efficacy and safety of ARR.

## 1. Introduction

Asari Radix et Rhizoma (ARR), an important traditional Chinese medicine, is derived from the dried root and rhizome of *Asarum heterotropoides* Fr. Schmidt var. *mandshuricum* (Maxim.) Kitag. (AHM), *A. sieboldii* Miq. var. *seoulense* Nakai, or *A. sieboldii* Miq., and has been used to dispel wind, dissipate cold, and relieve pain [1].

Traditionally, ARR has been mostly used in the form of decoction and powder preparations in Chinese medicine clinical practice [1]. ARR decoction is used to treat headache, arthralgia, arrhythmia, rhinitis, and other conditions, especially for “wind–cold” headache and migraine, and ARR powder is also used clinically to alleviate pain and rhinitis, mainly to treat headache [2]. ARR decoction has a larger safe dose in clinical practice, whereas the dose of powder is strictly restricted; even the “toxicity” of ARR was recorded in Zheng Lei Ben Cao (Shen-Wei Tang, Northern Song dynasty, 1108 AD) and Ben Cao Gang Mu (Shi-Zhen Li, Ming dynasty, 1578 AD).

Volatile oils, lignans, and alkamides are considered to be the major constituents related to the pharmacological activity of ARR [3,4,5,6]. During the past few years, several studies analyzing the volatile oils, lignans, and alkamides in ARR by gas chromatography–mass spectrometry (GC–MS) [7], high–performance liquid chromatography (HPLC) [8], and ultra–performance liquid chromatography (UPLC) [9,10] have been reported, and these constituents can be used as marker compounds for the quality control of ARR.

The analysis of constituents of ARR in vivo was aimed at the volatile or nonvolatile constituents. Headspace solid-phase microextraction gas chromatography mass spectrometry (HS–SPME–GC–MS) was adopted for the quantitative study of seven major volatile compounds in mouse brain, liver tissues, and blood after an intragastric administration of ARR (AHM) [11]. A total of 48 absorbed constituents were identified by HS–SPME–GC–MS and high–performance liquid chromatography atmospheric pressure chemical ionization ion trap time-of-flight multistage mass spectrometry (HPLC–APCI–IT–TOF–MS^n^) in rabbit plasma and cerebrospinal fluid after an intranasal administration of ARR (AHM) ethyl acetate extraction [12]. An LC/MS/MS method was applied in a pharmacokinetic study of ARR (AHM) extract after it was orally administered to rats, and two epimeric furofuran lignans (sesamin and asarinin) were detected simultaneously in the plasma [13]. To date, the metabolic research on the constituents of ARR has not been sufficient. The metabolic characteristics of the constituents of ARR after oral administration of decoction and powder and the distribution of these constituents in tissues and organs have not been studied, which might be a key to evaluating the efficacy and safety of ARR in different administration forms.

This research aims to describe the difference in the relative content and distribution of absorbed VOCs between ARR (AHM) powder- and decoction-treated groups, to compare their absorption and distribution characteristics in vivo. The results of this study will provide a reference for quality control and evaluation of the efficacy and safety of ARR.

## 2. Results and Discussion

### 2.1. Identification of ARR VOCs In Vivo in Rats

**Figure 1:**
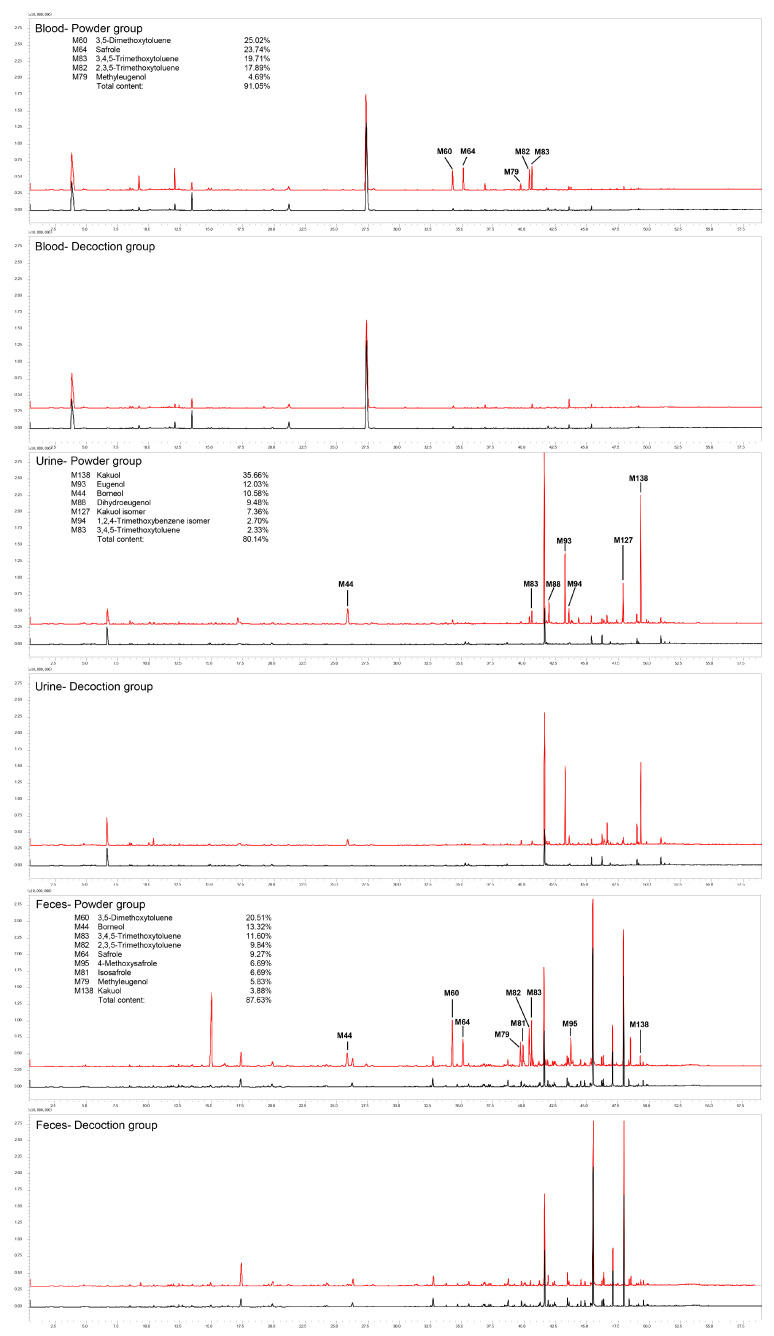
Total ion chromatograms (TICs) of compounds identified in blood, urine, and feces of rats after oral administration of ARR powder or decoction by HS–SPME–GC–MS. Contents were calculated from extracted ion chromatography (EIC) according to area generalization method. Red line: treatment group; black line: control group.

**Figure 2:**
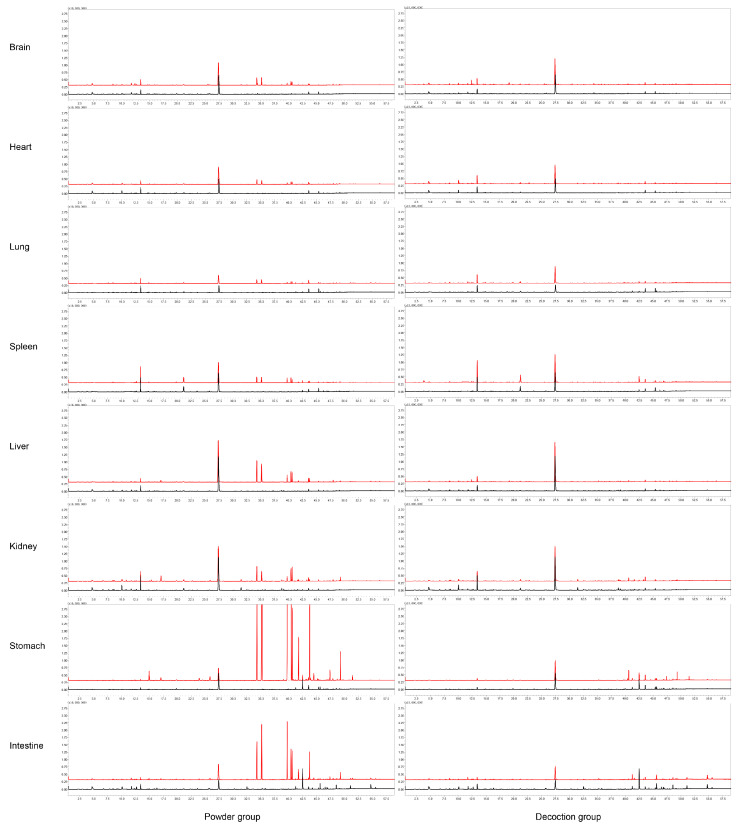
Total ion chromatograms (TICs) of compounds identified in brain, heart, lung, spleen, liver, kidney, stomach, and small intestine of rats after oral administration of ARR powder or decoction by HS–SPME–GC–MS. Red line: treatment group; black line: control group.

A total of 153 VOCs of ARR were detected in this study. Based on the structure, the 153 VOCs could be divided into three main categories: 63 terpenoids and their substitutes (including monoterpenes and sesquiterpenes), 7 alkanes and alkenes (including straight chains, branched chains, and ring structures), and 83 aromatic compounds. Among the 153 VOCs, 101 could be identified as original constituents of ARR (98 in the powder-treated group and 43 in the decoction-treated group), and 15 were identified as the metabolites of ARR constituents (12 in the powder-treated group and 6 in the decoction-treated group). The metabolic reactions were mainly phase I reactions (oxidation and reduction), and there were only two cases of phase II metabolic reactions (methylation and esterification). The remaining unclassified 37 compounds were identified in the ARR-treated groups (24 in the powder-treated group and 25 in the decoction-treated group), but could not be found in either the control group or the ARR sample itself, suggesting that they might be metabolites of constituents or endogenous substances generated after the administration of ARR, and more evidence was required to determine their source. Of the 153 VOCs identified, 131 were first reported in the rats after oral administration of ARR (79 original constituents, 15 metabolites, and 37 unclassified compounds, or 79/15/37, as designated hereafter). A list of all compounds detected in this study and chromatograms of all samples are shown in Table 1 and Figure 1 and Figure 2. The structures of all identified compounds are shown in Supplementary Materials Figures S1 and S2.

After oral administration of ARR powder, a total of 134 VOCs (98/12/24) were detected in the feces, urine, and eight organs of rats, most of which were identified in the urine (40/9/19), followed by the stomach (59/1/2), feces (49/1/2), kidney (36/4/2), small intestine (33/1/0), liver (27/1/0), blood (24/0/0), spleen (17/0/0), brain (15/0/1), heart (14/0/1), and lung (14/0/1).

Similarly, 74 VOCs (43/6/25) were detected in the decoction-treated group. There were 48 (26/5/17) VOCs in urine, 32 (28/0/4) in stomach, 26 (22/1/3) in kidney, 20 (19/0/1) in feces, 19 (17/0/2) in small intestine, 18 (15/1/2) in liver, 11 in (11/0/0) blood, 9 (9/0/0) in spleen, 7 (6/0/1) in lung, 5 (5/0/0) in brain, and 5 (5/0/0) in heart.

### 2.2. Distribution of ARR VOCs In Vivo in Rats

**Figure 3:**
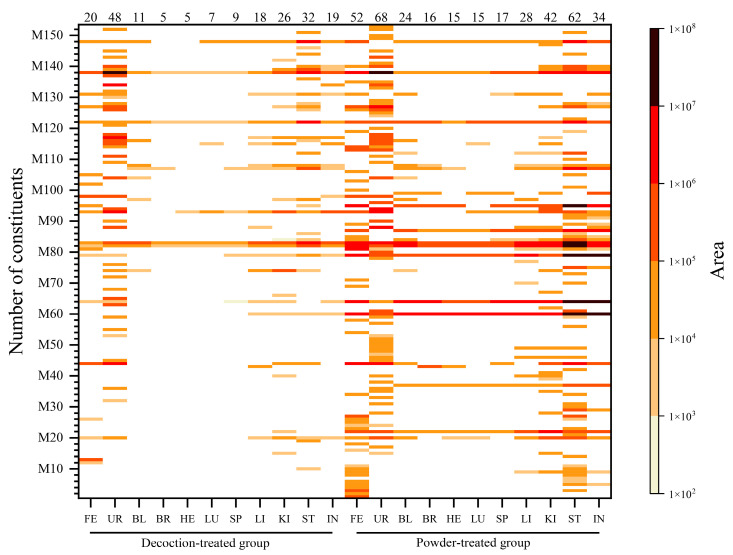
Heatmap of identified compounds. Peak area of compounds identified in feces, urine, blood, and eight organ samples (brain, heart, lung, spleen, liver, kidney, stomach, and small intestine) are illustrated using heatmaps, in which the color shade (shown in the right of the ordinate) indicates the peak area size of a compound. Darker band indicates a larger peak area. Serial numbers of compounds (shown on the ordinate) were sorted by the retention time of all compounds identified in each sample, and the larger the compound number, the higher the polarity. Numbers of compounds identified in each sample are shown above the horizontal axis. FE: feces; UR: urine; BL: blood; BR: brain; HE: heart; LU: lung; SP: spleen; LI: liver; KI: kidney; ST: stomach; IN: small intestine.

In this research, a total of 153 VOCs was detected. Among them, 84 VOCs (44/12/28) were identified in the urine, followed by 70 (65/1/4) in the stomach, 56 (52/1/3) in the feces, 43 (36/3/4) in the kidney, 36 (33/1/2) in the small intestine, 32 (29/1/2) in the liver, 24 (24/0/0) in the blood, 17 (17/0/0) in the spleen, 15 (14/0/1) in the brain, 15 (14/0/1) in the heart, and 15 (14/0/1) in the lung. Moreover, to the best of our knowledge, except for estragole, methyleugenol, 2,3,5-trimethoxytoluene (2,3,5-TMT), 3,4,5-trimethoxytoluene (3,4,5-TMT), sarisan, 3,5-dimethoxytoluene (3,5-DMT), and safrole were reported to be distributed in the brain and liver of mice [11], and 26 VOCs were identified in the plasma and cerebrospinal fluid of ARR-treated rabbits, 22 of which were also identified in this study [12]; the distributions of the other 131 constituents (79/15/37) in eight rat organs (heart, liver, spleen, lung, kidney, brain, stomach, and small intestine) and blood, urine, and feces after oral administration of ARR were reported for the first time. The distribution of all 153 identified VOCs (101/15/37) are shown in Figure 3, Table 2 and Table 3, and Supplementary Materials Table S1.

#### 2.2.1. Distribution of VOCs in ARR Powder-Treated Group

A total of 134 VOCs (98/12/24) of ARR were identified in the powder-treated group. Twenty-four original constituents can be absorbed into the blood, including M20 (*dl*-camphor), M22 (eucarvone), M37 (estragole), M44 (borneol), M60 (3,5-DMT), M64 (safrole), M79 (methyleugenol), M82 (2,3,5-TMT), M83 (3,4,5-TMT), M107 (elemicin), and M138 (kakuol). Fifteen of these 24 original constituents can be distributed to the brain: eucarvone, estragole, 3,5-DMT, safrole, methyleugenol, 2,3,5-TMT, 3,4,5-TMT, M87 (*E*-isocroweacin), M95 (4-methoxysafrole), M99 (*p*-methoxypropiophenone), elemicin, M108 (3,4,5-TMT isomer), M122 (3,4-methylenedioxypropiophenone), kakuol, and M148 (2′,4′-dimethoxy-3′-methylpropiophenone). Except for M99 and M108, the other 13 constituents identified in the brain were also found in the heart. Additionally, M20, identified in the heart, was not detected in the brain. Because the main pharmacological effect of ARR is analgesia and the toxicity of ARR powder is mainly respiratory paralysis, it is speculated that the constituents that can be distributed to the blood, lungs, and brain might be the main active VOCs of ARR.

Similarly, 27 and 36 original constituents of ARR were found in the liver and kidney, respectively. Moreover, as the main digestive organs, 59 and 33 original constituents were identified in the stomach and small intestine, and the peak areas of the main identified constituents (3,5-DMT, 2,3,5-TMT, 3,4,5-TMT, methyleugenol, and safrole) were higher than those in the other organs. The ratio between the peak area of 3,5-DMT in the stomach and in the brain, heart, lung, spleen, liver, and kidney was 14.2, 24.2, 27.7, 18.9, 5.0, and 7.6, respectively. For the small intestine, the ratio was 5.3, 9.0, 10.3, 7.0, 1.8, and 2.8, respectively. The distribution of 2,3,5-TMT, 3,4,5-TMT, methyleugenol, and safrole in the different organs showed the same pattern. The peak areas of the main identified constituents are shown in Table 4.

The main constituents found in the feces, 3,5-DMT, borneol, 3,4,5-TMT, 2,3,5-TMT, safrole, 4-methoxysafrole, M81 (isosafrole), methyleugenol, kakuol, and M98 (thymol), were all original constituents of ARR, and the sum of peak areas of these constituents account for more than 90% of the total peak area.

Kakuol, M93 (eugenol), borneol, M88 (dihydroeugenol), M127 (kakuol isomer), M94 (1,2,4-TMT isomer), and 3,4,5-TMT were the main VOCs detected in the urine, and the sum of their peak areas accounts for more than 80% of the total peak area. Among them, M88 and M94 were not original constituents and were speculated to be metabolites of eugenol and 2,3,5-TMT.

In general, the polarity of the constituents identified in the feces was lower than that detected in the urine. Monoterpenes were detected in the feces rather than urine, including M1 (α-pinene), M2 (camphene), M3 (β-pinene), M4 (sabinene), M5 (3-carene), M6 (β-myrcene), M8 (limonene), and M11 (terpinolene). It was assumed that these low-polarity constituents remained in the residue of the ARR powder and were excreted directly through the digestive tract.

A total of nine VOCs could be detected in all samples of feces, urine, blood, and eight organs of the rats after oral administration of ARR powder: 2,3,5-TMT, 3,4,5-TMT, methyleugenol, safrole, eugenol, eucarvone, 3,5-DMT, 3,4-methylenedioxypropiophenone, and kakuol.

Additionally, 12 metabolites were identified in the powder-treated group. Most of the metabolites were detected in the urine (9/12) and kidney (4/12). As a reduced metabolite of eugenol, dihydroeugenol was more widely distributed in the urine, liver, kidney, and small intestine than the other metabolites. No metabolite of ARR was found in the blood. The distribution of metabolites is shown in Table 3.

#### 2.2.2. Distribution of VOCs in Decoction-Treated Group

A total of 43 original constituents and six metabolites of ARR were identified in the decoction-treated group. Compared to the powder-treated group, the peak areas of main constituents in the decoction-treated group (2,3,5-TMT, 3,4,5-TMT, kakuol, and 3,4-methylenedioxypropiophenone) were significantly reduced. For example, the ratio of the peak area of 2,3,5-TMT in the samples between the two groups was 629 for feces, 22 for urine, 59 for blood, 103 for brain, 79 for heart, 49 for lung, 104 for spleen, 73 for liver, 46 for kidney, 64 for stomach, and 251 for intestine. The main absorbed constituents of the powder-treated group (3,5-DMT, safrole, and methyleugenol) were not detected in the blood, brain, heart, or lung in the decoction-treated group. Moreover, 3,5-DMT was not detected in the urine or feces. The peak areas and the ratio between the main absorbed constituents between groups are shown in Table 4.

Eleven original constituents were identified in the blood: thymoquinone, 2,3,5-TMT, 3,4,5-TMT, M97 (2,4,5-trimethoxybenzoic acid), M104 (piperonal), elemicin, M108 (3,4,5-TMT isomer), M116 (3,4-methylenedioxyacetophenone), M122 (3,4-methylenedioxypropiophenone), kakuol, and M148 (2′,4′-dimethoxy-3′-methylpropiophenone). Five constituents were detected in the brain: 2,3,5-TMT, 3,4,5-TMT, elemicin, 3,4-methylenedioxypropiophenone and kakuol. Six metabolites were detected in the liver, kidney, and urine: M15 (*cis*-limonene oxide), M36 (*l*-pinocarveol), M47 (α-cyclogeraniol), M88 (dihydroeugenol), M132 (piperonol), and M137 (methoxyeugenol). No metabolite of ARR was found in the blood, feces, or other organs. In general, lower numbers and peak areas of VOCs were detected in the decoction-treated group, which suggests that many VOCs with low polarity and strong volatility were lost during boiling water decoction [14]. Additionally, it is interesting that safrole, which is considered to be a toxic constituent of ARR, was not distributed to the blood, brain, lungs, or heart in the decoction-treated group. It was suggested that after oral administration of ARR decoction, the levels of safrole and/or other potential toxic constituents in vivo are very low, and they are no longer distributed to important organs, thus ARR can be safely used in higher doses after decoction than before or in powder form.

#### 2.2.3. Distribution of Main Compounds Identified In Vivo in Rats

The peak areas of all identified compounds of ARR in the urine, feces, blood, and eight organs (brain, heart, lung, spleen, kidney, liver, stomach, and small intestine) were calculated and sorted, and the constituents with max peak areas were selected. In the powder-treated group, the five main constituents in the blood and organ samples were 3,5-DMT, 2,3,5-TMT, 3,4,5-TMT, methyleugenol, and safrole. The main compounds in the urine were kakuol, eugenol, borneol, dihydroeugenol, and M127 (kakuol isomer). The sum of their peak areas accounts for at least 75% of the total peak areas (calculated by normalization areas) of all compounds identified in each sample. In the feces, 35-DMT, borneol, 3,4,5-TMT, 2,3,5-TMT, and safrole were the main constituents detected, and their peak area accounts for 65% of the total peak areas. Except dihydroeugenol, which is a metabolite, these compounds were original constituents of ARR: 3,5-DMT, 2,3,5-TMT, 3,4,5-TMT, methyleugenol, safrole, kakuol, eugenol, borneol, and M127.

In the decoction-treated group, 3,4,5-TMT, 2,3,5-TMT, and kakuol were the main constituents identified in the brain, heart, lung, spleen, and liver, with a total content higher than 65% in each sample. Especially, the content of 3,4,5-TMT was higher than 50% in the brain, heart, lung, and spleen. In the kidney, stomach, and small intestine, 3,4,5-TMT and kakuol had the highest content, accounting for 34.0% and 19.2% in the kidney, 23.7% and 30.0% in the stomach, and 17.3% and 28.4% in the intestine, respectively; however, the content of 2,3,5-TMT decreased to 2%–3% in these three organs. Eugenol replaced 2,3,5-TMT as the main constituent in the kidney and intestine (14.2% and 15.3%), and M148 (2′,4′-dimethoxy-3′-methylpropiophenone) became a substitute in the stomach (17.4%). In the blood, 3,4,5-TMT, 2′,4′-dimethoxy-3′-methylpropiophenone, and 2,3,5-TMT were the main absorbed constituents, with contents of 65.5%, 9.5%, and 6.9%, respectively. Kakuol, eugenol, and borneol were the main constituents identified in the urine, with contents of 37.79%, 23.67%, and 6.67%, respectively. In the feces, the main constituents were kakuol (41.2%), borneol (31.0%), and thymol (6.5%).

The ratios of peak areas of 3,5-DMT, safrole, methyleugenol, 2,3,5-TMT, 3,4,5-TMT, eugenol, and kakuol between the powder- and decoction-treated groups are shown in Table 4, and the values of the peak area of all identified compounds are shown in Supplementary Materials Tables S2–S23.

### 2.3. Metabolism of VOCs of ARR

In this research, a phase I reaction (oxidation and reduction) was the main type of metabolic reaction of VOCs after oral administration of ARR powder or decoction, and only two cases of a phase II reaction (methylation and esterification) were found. One possible explanation is that the products of phase II metabolites are generally non-volatile and could not be detected by the GC–MS equipment with HS–SPME. To detect the products of phase II metabolites, other analytical techniques are required, such as LC–MS, or GC–MS analysis after derivation.

#### 2.3.1. Metabolites of the Oxidation Reaction

The double bond of M8 (limonene) was oxidized to form the epoxy bond and converted to M15 (*cis*-limonene oxide) [15]. M33 (*l*-menthol) was oxidized by cytochrome P450s, and M24 (isopulegol) was produced. Hydroxylation was found in the carbon atom adjacent to the double bond of M3 (β-pinene), and M36 (*l*-pinocarveol) was formed [16].

M64 (safrole) was oxidized to form M141 (3,4-methylenedioxypheny-1-propanal) and M149 (1-hydroxy-2-(prop-2-enyl)-4,5-methylenedioxybenzene), and M96 (*m*-eugenol) was formed after the breaks and methylation of methylenedioxybenzene [17,18,19]. M11 (terpinolene) was transformed into M42 (α-terpineol acetate) after oxidation and esterification.

Metabolites M132 (piperonol) and M137 (methoxyeugenol) were detected only in the decoction-treated group, and their original constituents were presumed to be M104 (piperonal) [20] and M93 (eugenol).

#### 2.3.2. Metabolites of the Reduction Reaction

**Figure 4:**
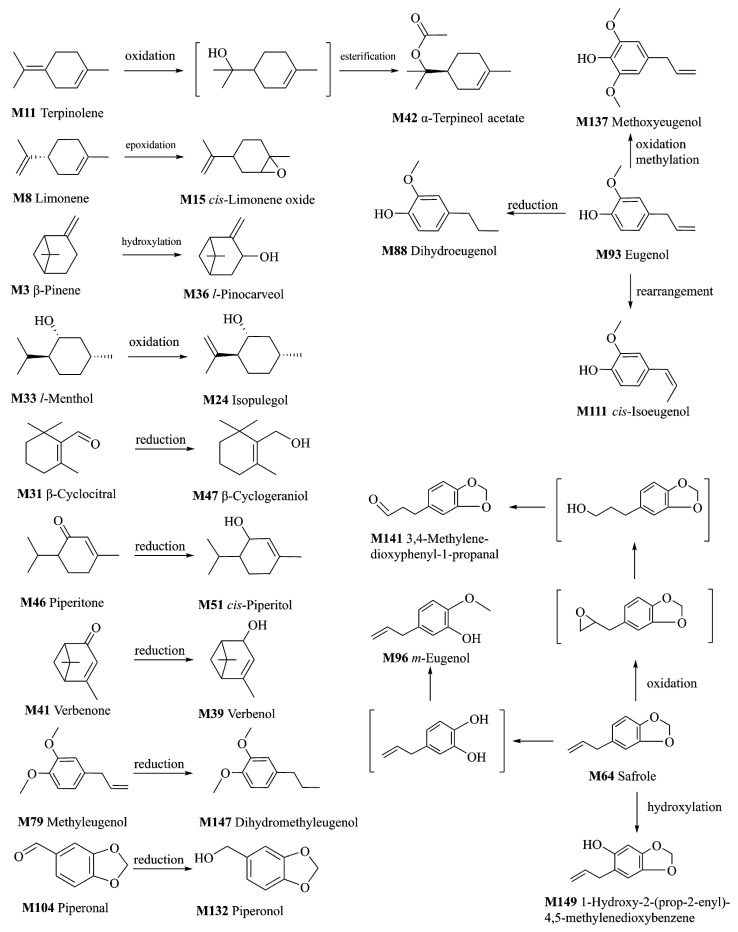
Proposed metabolic pathways of some volatile constituents of ARR.

The carbonyl groups of M31 (β-cyclocitral), M46 (piperitone), and M41 (verbenone) were reduced to hydroxyl groups to form M47 (β-cyclogeraniol), M51 (*cis*-piperitol), and M39 (verbenol). The double bonds of M93 (eugenol) and M79 (methyleugenol) were reduced to M88 (dihydroeugenol) and M147 (dihydromethyleugenol) [21]. The metabolites and their metabolic reactions are shown in Figure 4.

### 2.4. Review of the Bioactivity and Acute Toxicity of Main Absorbed Constituents

The bioactivity and acute toxicity of the main absorbed constituents (3,5-DMT, 2,3,5-TMT, 3,4,5-TMT, methyleugenol, safrole, eugenol, and kakuol) were reviewed and the results were as follows.

It was found that 3,4,5-TMT has anti-inflammatory activity in vitro through the pathway of suppressing lipopolysaccharide-induced NO production [22]. It was also found that 3,5-DMT has a sedative effect [23]. Methyleugenol has anti-inflammatory [24], analgesic [25], spasmolytic [26], antiallergy [27], cardioprotective [28], and anticonvulsive [29] activity. Safrole, reported to be a toxic constituent in ARR, is carcinogenic and its metabolites can inhibit the respiratory center [30]. Eugenol can be used in the treatment of diseases associated with oxidative stress and inflammatory responses through the inhibition of enzymes and oxidative processes [31].

As mentioned at the beginning of this paper, in traditional Chinese medicine, the toxicity of ARR powder is a matter worthy of attention. It was reported that the median oral lethal dose (LD_50_) of powder, decoction, and volatile oil of ARR (AHM root) in mice was 4.8 g/kg, 240 g/kg, and 2.53 mL/kg, respectively [32]. For its very small LD_50_, the volatile oil is considered to be the substance that causes the toxicity of ARR powder. Since the decoction can be used safely in a larger dose, whereas the dose of powder is strictly restricted, and the acute toxicity of ARR decreases significantly after boiling water decoction, we considered that the difference in the absorbed compounds between the powder- and decoction-treated groups should be the main factor responsible for the toxicity of ARR powder. In this research, 3,5-DMT, safrole, and methyleugenol were detected as the main constituents of ARR in the power-treated group, which distributed into the blood and all eight organ samples (brain, heart, lungs, spleen, liver, kidney, stomach, and small intestine). In contrast, in the decoction-treated group, 3,5-DMT, safrole, and methyleugenol were not found in blood and organs related to ARR toxicity (brain, heart, and lung). The peak area ratios of 3,5-DMT, safrole, and methyleugenol in these organs between the two groups were as follows: liver—4198, 4062, and 397; kidney—2430, 1367, and 173; small intestine—6101, 6411, and 1793. The results are shown in Table 4.

On the other hand, 3,5-DMT, methyleugenol, and safrole have a lower polarity and stronger volatility and are easier to lose during decoction. It was reported that after 1 h decoction, the amount of safrole decreased by more than 92%, resulting in the equivalent of no more than 0.20 mg/g safrole remaining in the aqueous extract. Similarly, the content of methyleugenol decreased by more than 60% [14]. Therefore, we can speculate that methyleugenol, safrole, and 3,5-DMT may be the main toxic constituents in the volatile oil of ARR.

At present, there are a few reports on the acute toxicity of safrole, and its LD_50_ was calculated as follows: rat (oral)—1950 mg/kg; mouse (oral)—2350 mg/kg; mouse (subcutaneous)—1020 mg/kg [33]. The LD_50_ of methyleugenol was also evaluated, and the values were: rat (oral)–1179 mg/kg; mouse (intraperitoneal)—540 mg/kg; mouse (intravenous)—112 mg/kg [33], and mouse (intravenous) > 640 mg/kg [34]. The LD_50_ of 3,5-DMT has not been reported.

In addition, considering that methyleugenol and safrole are not highly toxic compounds and their oral LD_50_ values are reported to be 1179 (rat) and 2350 mg/kg (mouse), respectively, the acute toxicity of other compounds detected in the blood of the powder-treated group were also investigated. The results show that these compounds also showed low oral toxicity, and their LD_50_ values are shown in Table 5.

Based on the above discussion, we speculate that the toxicity of ARR powder might be derived from the combined effect of a large number of such compounds which can be absorbed with low or very low contents and have similar metabolic characteristics with the main absorbed constituents, rather than a single or a few compounds, and an additive toxic effect might be the mechanism. Further research on the toxic constituents of ARR is necessary.

## 3. Experiment

### 3.1. Chemicals and Reagents

Limonene (Lot: MKBH7774V), β-pinene (Lot: BCBH3864V), and estragole (Lot: MKBN4968V) were purchased from Sigma (St. Louis, MO, USA). L-Borneol (Lot: EPH8L-QQ), eucarvone (Lot: F1101-DLFG), and methyleugenol (Lot: PH3YH-MG) were purchased from TCI (Tokyo, Japan). 3,5-Dimethoxytoluene (Lot: 10099004) was purchased from Alfa Aesar (Heysham, UK). Safrole (10 mg in 200 μL ethanol; Lot: 0452680-11) was purchased from Cayman Chemical (Ann Arbor, MI, USA). 3,4,5-Trimethoxytoluene (Lot: 19923) was purchased from Aladdin Industrial (Shanghai, China). Elemicin (Lot: SY018605) was purchased from Accela ChemBio (Shanghai, China). 3,4-(Methylenedioxy)-propiophenone (Lot: M38410CCR0) was purchased from Heowns Biochem (Tianjin, China). Kakuol (Lot: Y19J6H1) was purchased from Shanghai Yuanye Bio-Technology (Shanghai, China). Eugenol (Lot: FD050202) was purchased from Sun Chemical Technology (Shanghai, China). β-Asarone (Lot: BBP01604) was purchased from Biobiopha (Kunming, China). 2,3,5-Trimethoxytoluene was synthesized by the authors and the structure was identified by MS and NMR, and the purity was over 98% (GC–MS, area normalization method). A mixture of *n*-alkanes (C_7_–C_30_, Lot: LC13543V) to calculate the retention indices (RIs) of all volatile compounds was purchased from Sigma (St. Louis, MO, USA). Water was obtained from a Milli-Q water purification system (Millipore, Bedford, MA, USA). All other reagents and chemicals were analytical grade.

### 3.2. Plant Material

The plant samples of ARR (No. 20140807-1) were collected by the authors from Xinbin, Liaoning, China. The roots and rhizomes of the plants were washed and dried in the shade. All samples were identified by Professor Shao-Qing Cai as *Asarum heterotropoides* Fr. Schmidt var. *mandshuricum* (Maxim.) Kitag. (AHM). The samples were stored in airtight containers at room temperature. The voucher sample (No. 20140807-1) was deposited in the Herbarium of Pharmacognosy, School of Pharmaceutical Sciences, Peking University (China).

### 3.3. Sample Preparation

#### 3.3.1. Dosage of Administration

According to the Pharmacopoeia of China (2015 edition), the appropriate dosage of ARR for decoction administration is 1–3 g crude drug per dose, and for powder administration it is no more than 1 g each time. In this study, the dosages of ARR decoction and powder were set at 10 times the upper limit of the recommended dosage of the pharmacopeia, namely 30 and 10 g, respectively.

#### 3.3.2. Decoction of ARR

The roots and rhizome of 100 g of AHM (No.20140807-1) were cut into pieces 1 cm in length and put into a decoction pot (4 L; Feilu Waner Electric Co., Ltd., Guangdong, China), and immersed in 800 mL deionized water for 30 min, then boiled for 50 min. Two layers of gauze filter were used to obtain ARR decoction. Then, 600 mL of deionized water was added to the residue and boiled for 50 min again. The mixed decoction was concentrated to 0.4 g/mL by evaporation in a rotating evaporator (Büchi B-220, Flawil, Switzerland) at low temperature (<40 °C). The ARR decoction solution was stored in a refrigerator at −80 °C until use.

#### 3.3.3. Suspension Solution of ARR Powder

The roots and rhizome of 100 g of AHM (No. 20140807-1) were pulverized and sieved through 120 mesh (mesh size 125 μm). The powder was suspended in a 0.4% sodium carboxymethyl cellulose solution to prepare a powder suspension solution of ARR (0.131 g/mL). The ARR powder suspension solution should be prepared on the day of administration.

### 3.4. HS-SPME-GC–MS Analysis

The analyses were performed on a Shimadzu GC/MS QP–2010 Ultra system (Kyoto, Japan) equipped with an AOC–5000 autosampler. Chromatographic separations were conducted on a DB–WAXms capillary column (30 m × 0.25 mm, 0.25 μm film thickness) (Agilent, Wilmington, DE, USA).

The column temperature was programmed as follows: initial oven temperature 40 °C, then raised to 100 °C at a rate 5 °C/min and held for 10 min, 5 °C/min to 110 °C and held for 5 min, 5 °C/min to 190 °C, and finally 10 °C/min to 230 °C and held for 10 min. The total run time was 59 min. The cool-down period was about 8 min before the next injection. High-purity helium was used as the carrier gas at a flow rate of 1.2 mL/min. The splitless injection mode was used, and the injection temperature was 230 °C. MS detection was performed in electron ionization mode at −70 eV, and the mass spectrometer was operated in scan mode over a mass range of *m*/*z* 35–500 at a rate of 0.3 s/scan. The ionization source and interface temperatures were 200 and 230 °C, respectively.

HS–SPME was selected as the extraction method, because in the headspace mode, the fibers can avoid touching the biological samples directly, which can protect the fibers and prolong the service life. The optimization of HS–SPME conditions was performed based on our previous research results [11,12], and a SUPELCO DVB/CAR/PDMS fiber (Bellefonte, PA, USA) was selected. Sample vials (10 mL; GL Science, Kyoto, Japan) were incubated at 70 °C for 5 min prior to microextraction, and the adsorption time was 30 min at 70 °C. After adsorption, the fiber was withdrawn and transferred into the injection port of the GC. The temperature of the injection port was 230 °C and the desorption time was 3 min. After the chromatographic run, the fiber was conditioned for 1 min at 250 °C for the next HS–SPME cycle.

### 3.5. Animals and Drug Administration

Male Sprague-Dawley (SD) rats weighing 250 ± 20 g were obtained from the Experimental Animal Center of Peking University Health Science Center (Beijing, China). All animal experiments were performed in accordance with the Guideline for Animal Experimentation of Peking University Health Science Center (No: SYXK-2016-0041), and the protocol was approved by the Animal Ethics Committee of the institution (Approval No. LA2011-76). Eighteen rats were randomly divided into 3 groups (6 animals each): powder-treated (group I), decoction-treated (group II), and control (group III). The rats were maintained in metabolic cages (type DXL-DL; Suzhou Fengshi Laboratory Animal Equipment Co. Ltd., Suzhou, China) and acclimatized to the facilities for 5 days prior to experiments. They were housed under standard environmental conditions with a temperature of 25 ± 2 °C and 30%–70% humidity under a 12 h light–dark cycle and allowed free access to drinking water and a standard no-residue diet.

ARR powder was suspended in 0.4% carboxyl methyl cellulose sodium (CMC-Na) solution and orally administered at a dose of 2.0 mL/250 g body weight (group I), ARR decoction was orally administered at a dose of 2.0 mL/250 g body weight (group II), and control group rats were orally administered 0.4% CMC-Na solution at 2.0 mL (group III). All rats were dosed twice per day at a 12 h interval (09:00 and 21:00) for 5 days, and their urine and feces were measured and cleaned up.

### 3.6. Sample Collection and Preparation

Urine and feces samples from rats were collected 1 h after the last administration (on day 5). Urine samples from the same group, 5 mL per rat, were merged into one sample and the mixture was centrifuged at 12,000 rpm and 4 °C for 30 min. Subsequently, a sample solution containing 20% (w/v) NaCl was prepared by adding 0.1 g NaCl to 500 μL of the supernatant. The sample solution was put in a sample vial and stored in a refrigerator at −80 °C until HS–SPME–GC–MS analysis.

Feces samples from the same group were merged into one sample and weighed. Then, these samples were ground in four-fold volume (volume/mass wet weight) deionized water and a sample solution containing 20% (*w*/*v*) NaCl was prepared by adding 0.1 g NaCl to 500 μL of the suspension. The sample solution was put in a sample vial and stored in a refrigerator at −80 °C.

Blood samples were collected in heparinized tubes using a heart puncture technique under anesthesia (10% chloral hydrate, intraperitoneal injection, 0.3 mL/100 g body weight) 1 h after the last administration. Samples from the same group, 1 mL per rat, were merged into one sample. A sample solution containing 20% (*w*/*v*) NaCl was prepared and stored at −80 °C until analysis.

After the collection of blood samples, eight organs (brain, liver, spleen, lungs, kidneys, heart, stomach, and small intestine) of the rats in each group were rapidly removed and flushed with 4 °C deionized water, dried with filter paper, and weighed. The same organ samples from each group were merged into one sample and processed using a homogenizer (T10 Basic ULTRA-TURRAX, IKA, Staufen, Germany) following suspension in a four-fold volume (volume/mass organ wet weight) of 4 °C deionized water. Then, a sample solution containing 20% (w/v) NaCl was prepared by adding 0.1 g NaCl to 500 μL of the suspension. All samples were prepared and stored at −80 °C until analysis.

### 3.7. Identification of Compounds

The difference in chromatographic peaks between the control and ARR-treated groups was found first. The NIST 11 library (version 2011; National Institute of Standards and Technology, Gaithersburg, MD, USA) embedded in the GCMSsolution software of the GC–MS workstation (version 2.71; Shimadzu, Kyoto, Japan) was used for the preliminary identification of the mass spectra of the target compounds. The retention index (RI) of chromatographic peaks was calculated for all VOCs using the retention data of linear *n*-alkanes C_7_–C_30_, and the RI values from the literature were also retrieved from the NIST Chemistry WebBook (NIST Standard Reference Database Number 69, http://webbook.nist.gov/chemistry/). Compounds could be identified if the RI calculated from experiments matched the RI retrieved from the NIST Chemistry WebBook. Furthermore, reference compounds were used in the identification by comparing the retention time and mass spectra with the target compounds.

As stated previously, the identification levels of VOCs in this study were as follows:

Level 1: Compounds were identified by comparing their retention time and mass spectra with those of reference compounds.
Level 2: Compounds were identified by comparing their RI and mass spectra with those of the literature.
Level 3: Compounds were identified by searching mass spectra in NIST 11.

After identification, the peak areas were calculated by extraction ion chromatography (EIC) of the compounds, and the extraction ions, which represented the molecular ion peak of the compounds or the base peak of the mass spectra, are shown in Supplementary Materials Tables S2–S23. Based on the peak area of VOCs, further analysis was conducted of the distribution of VOCs in the feces, urine, blood, and eight organ samples of the rats in each group.

## 4. Conclusions

This study describes the metabolite profile and distribution of VOCs of ARR. An HS–SPME–GC–MS method was established, and a total of 153 VOCs were tentatively identified in rats in vivo, 101 of which were original constituents of ARR and 15 were metabolites, the metabolic reactions of which were mainly phase I (oxidation and reduction), with only two cases of phase II (methylation and esterification). Of the 153 VOCs identified, 131 are reported for the first time in rats after oral administration of ARR. The detected VOCs were wildly distributed to the feces, urine, blood, and organ tissues (brain, heart, lung, spleen, liver, kidney, stomach, and small intestine) in the rats after oral administration of ARR decoction or powder. The original constituents were found with the highest numbers in the stomach (65), followed by feces (52), urine (44), kidney (36), small intestine (33), liver (29), blood (24), spleen (17), brain (14), heart (14), and lung (14). Moreover, the original constituents were found with much higher numbers in the powder-treated group (98) than the decoction-treated group (43). Most metabolites were detected in the urine (12/15), whereas only 1 metabolite was found in the feces (1/15). In addition, the kidney was the main distribution organ of metabolites, with a total of four metabolites (4/15), compared to the liver, stomach, and small intestine, with one each. Methyleugenol, 3,5-DMT, 2,3,5-TMT, 3,4,5-TMT, and safrole were the main absorbed constituents in the powder-treated group, while 3,4,5-TMT, 2,3,5-TMT, and kakuol were the main absorbed constituents in the decoction-treated group. The different absorbed constituents between the two groups were mainly methyleugenol, safrole, and 3,5-DMT, which might play major roles in the acute toxicity of ARR powder. The results of this research provide a reference for evaluating the efficacy and safety of ARR.

## Figures and Tables

**Table 1:** Retention time (t_R_), Chemical Abstracts Service (CAS) number, molecular formula, and identification of 153 volatile compounds in rats in vivo after oral administration of Asari Radix et Rhizoma (ARR) powder or decoction by headspace solid-phase microextraction gas chromatography mass spectrometry (HS–SPME–GC–MS).

No. ^a^	Name of Compounds	CAS ^b^	t_R_ (min)	MW ^c^	Formula	RI ^d^	Identification ^e^
**M1**	α-Pinene	80-56-8	3.785	136	C_10_H_16_	1021	MS, RI
**M2**	Camphene	79-92-5	4.375	136	C_10_H_16_	1060	MS, RI
**M3**	β-Pinene	127-91-3	4.955	136	C_10_H_16_	1099	MS, RI, REF
**M4**	Sabinene	3387-41-5	5.175	136	C_10_H_16_	1110	MS, RI
**M5**	3-Carene	13466-78-9	5.700	136	C_10_H_16_	1135	MS, RI,
**M6**	β-Myrcene	123-35-3	6.080	136	C_10_H_16_	1154	MS, RI
**M7**	*d*-4-Carene	29050-33-7	6.405	136	C_10_H_16_	1170	MS, RI
**M8**	Limonene	138-86-3	6.830	136	C_10_H_16_	1191	MS, RI, REF
**M9**	Eucalyptol	470-82-6	7.095	154	C_10_H_18_O	1203	MS, RI
**M10**	*o*-Cymene	527-84-4	8.630	134	C_10_H_14_	1267	MS, RI
**M11**	Terpinolene	586-62-9	8.895	136	C_10_H_16_	1279	MS, RI
**M12**	Tridecane	629-50-5	9.217	184	C_13_H_28_	1293	MS, RI, REF
**M13**	Acetoin	513-86-0	9.328	88	C_4_H_8_O_2_	1298	MS, RI
**M14**	*p*-Cymenene	1195-32-0	12.885	132	C_10_H_12_	1433	MS, RI
**M15**	*cis*-Limonene oxide	13837-75-7	13.135	152	C_10_H_16_O	1441	MS, RI
**M16**	*cis*-4-Thujanol	15537-55-0	13.795	154	C_10_H_18_O	1462	MS, RI
**M17**	2-Nonen-4-one	32064-72-5	14.270	140	C_9_H_16_O	1477	MS, RI
**M18**	α-Copaene	3856-25-5	14.485	204	C_15_H_24_	1485	MS, RI
**M19**	Pentadecane	629-62-9	14.953	212	C_15_H_32_	1500	MS, RI
**M20**	*dl*-Camphor	76-22-2	15.305	152	C_10_H_16_O	1507	MS, RI
**M21**	1-Pentadecene	13360-61-7	16.070	210	C_15_H_30_	1523	MS, RI
**M22**	Eucarvone	503-93-5	17.060	150	C_10_H_14_O	1543	MS, REF
**M23**	*l*-Aristolene	6831-16-9	17.560	204	C_15_H_24_	1553	MS, RI
**M24**	Isopulegol	89-79-2	17.720	154	C_10_H_18_O	1556	MS, RI
**M25**	β-Copaene	18252-44-3	18.115	204	C_15_H_24_	1565	MS, RI
**M26**	Bornyl acetate	76-49-3	18.305	196	C_12_H_20_O_2_	1568	MS, RI
**M27**	1(10)-Aristolene	17334-55-3	18.760	204	C_15_H_24_	1577	MS, RI
**M28**	Fenchol	1632-73-1	18.920	154	C_10_H_18_O	1581	MS, RI
**M29**	Thymol methyl ether	1076-56-8	19.390	164	C_11_H_16_O	1590	MS, RI
**M30**	*l*-Aristolene isomer		19.650	204	C_15_H_24_	1596	MS
**M31**	β-Cyclocitral	432-25-7	20.510	152	C_10_H_16_O	1610	MS, RI
**M32**	Methyl benzoate	93-58-3	20.816	136	C_8_H_8_O_2_	1615	MS, RI
**M33**	*l*-Menthol	2216-51-5	21.220	156	C_10_H_20_O	1621	MS, RI
**M34**	δ-Guaiene	3691-11-0	21.535	204	C_15_H_24_	1626	MS, RI
**M35**	Umbellulone	24545-81-1	21.815	150	C_10_H_14_O	1630	MS, RI
**M36**	*l*-Pinocarveol	547-61-5	23.120	152	C_10_H_16_O	1651	MS, RI
**M37**	Estragole	140-67-0	23.905	148	C_10_H_12_O	1663	MS, RI, REF
**M38**	Isomenthol	490-99-3	24.295	156	C_10_H_20_O	1670	MS, RI
**M39**	Verbenol	473-67-6	24.570	152	C_10_H_16_O	1674	MS, RI
**M40**	Eucarvone isomer		24.990	150	C_10_H_14_O	1680	MS
**M41**	Verbenone	80-57-9	25.240	150	C_10_H_14_O	1684	MS, RI
**M42**	α-Terpineol acetate	80-26-2	25.300	196	C_12_H_20_O_2_	1685	MS, RI
**M43**	4-Ethylbenzaldehyde	4748-78-1	25.830	134	C_9_H_10_O	1693	MS, RI
**M44**	Borneol	507-70-0	25.875	154	C_10_H_18_O	1692	MS, RI
**M45**	Phellandral	21391-98-0	26.415	154	C_10_H_18_O	1703	MS, RI
**M46**	Piperitone	89-81-6	26.610	152	C_10_H_16_O	1706	MS, RI
**M47**	β-Cyclogeraniol	472-20-8	26.960	154	C_10_H_18_O	1711	MS
**M48**	*l*-Carvone	2244-16-8	27.270	150	C_10_H_14_O	1716	MS, RI
**M49**	Berbenone	80-57-9	28.190	150	C_10_H_14_O	1730	MS, RI
**M50**	*trans*-Piperitol	16721-39-4	29.165	154	C_10_H_18_O	1745	MS, RI
**M51**	*cis*-Piperitol	16721-38-3	29.485	154	C_10_H_18_O	1750	MS, RI
**M52**	Methyl benzeneacetate	101-41-7	29.955	150	C_9_H_10_O_2_	1758	MS, RI
**M53**	Myrtenol	515-00-4	31.910	152	C_10_H_16_O	1788	MS, RI
**M54**	6-Methyl-2-hepten-4-one	49852-35-9	32.060	126	C_8_H_14_O	1790	MS
**M55**	3,4-Dimethylbenzaldehyde	5973-71-7	32.061	134	C_9_H_10_O	1790	MS, RI
**M56**	Cuparene	16982-00-6	32.520	202	C_15_H_22_	1797	MS, RI
**M57**	*cis*-Sabinol	3310-02-9	32.645	152	C_10_H_16_O	1799	MS, RI
**M58**	Anethol	104-46-1	33.265	148	C_10_H_12_O	1815	MS, RI
**M59**	*cis*-Carveol	1197-06-4	34.050	152	C_10_H_16_O	1835	MS, RI
**M60**	3,5-Dimethoxytoluene	4179-19-5	34.260	152	C_9_H_12_O_2_	1840	MS, RI, REF
**M61**	*p*-Cymen-8-ol	1197-01-9	34.660	150	C_10_H_14_O	1851	MS, RI
**M62**	2,3-Dimethyldecahydronaphthalene	1008-80-6	34.995	166	C_12_H_22_	1859	MS
**M63**	Guaiacol	90-05-1	35.000	124	C_7_H_8_O_2_	1860	MS, RI
**M64**	Safrole	94-59-7	35.108	162	C_10_H_10_O_2_	1862	MS, RI, REF
**M65**	Benzyl alcohol	100-51-6	35.662	108	C_7_H_8_O	1877	MS, RI
**M66**	Verbenone isomer		36.215	150	C_10_H_14_O	1891	MS
**M67**	Verbenone isomer		36.360	150	C_10_H_14_O	1895	MS
**M68**	2-Phenylethanol	60-12-8	36.725	122	C_8_H_10_O	1906	MS, RI
**M69**	Agarospirol	1460-73-7	37.365	222	C_15_H_26_O	1928	MS
**M70**	3,4-Methylenedioxyanisole	7228-35-5	37.450	152	C_8_H_8_O_3_	1931	MS
**M71**	Isosafrole isomer		37.575	162	C_10_H_10_O_2_	1936	MS
**M72**	Creosol	93-51-6	38.108	138	C_8_H_10_O_2_	1954	MS, RI
**M73**	2-Allyl-1,4-dimethoxybenzene	19754-22-4	38.535	178	C_11_H_14_O_2_	1968	MS
**M74**	Thymoquinone	490-91-5	38.880	164	C_10_H_12_O_2_	1980	MS
**M75**	Methyleugenol isomer		39.055	178	C_11_H_14_O_2_	1986	MS
**M76**	6-Allyl-*o*-cresol	3354-58-3	39.435	148	C_10_H_12_O	2000	MS
**M77**	2,4-Dimethylanisole	6738-23-4	39.485	136	C_9_H_12_O	2002	MS
**M78**	*p*-Methoxybenzaldehyde	123-11-5	39.630	136	C_8_H_8_O_2_	2008	MS, RI
**M79**	Methyleugenol	93-15-2	39.715	178	C_11_H_14_O_2_	2011	MS, RI, REF
**M80**	*o*-Methylphenol	95-48-7	39.730	108	C_7_H_8_O	2012	MS, RI
**M81**	Isosafrole	120-58-1	39.925	162	C_10_H_10_O_2_	2020	MS, RI
**M82**	2,3,5-Trimethoxytoluene	38790-14-6	40.415	182	C_10_H_14_O_3_	2040	REF
**M83**	3,4,5-Trimethoxytoluene	6443-69-2	40.610	182	C_10_H_14_O_3_	2048	MS, RI, REF
**M84**	3,4,5-Trimethoxybenzoic acid	118-41-2	40.780	212	C_10_H_12_O_5_	2055	MS
**M85**	Globulol	51371-47-2	40.910	222	C_15_H_26_O	2061	MS, RI
**M86**	1,2,4-Trimethoxybenzene	135-77-3	41.430	168	C_9_H_12_O_3_	2083	MS, RI
**M87**	*E*-Isocroweacin	194609-21-7	41.755	192	C_11_H_12_O_3_	2096	MS
**M88**	Dihydroeugenol	2785-87-7	41.970	166	C_10_H_14_O_2_	2106	MS, RI
**M89**	Spathulenol	6750-60-3	42.110	220	C_15_H_24_O	2112	MS, RI
**M90**	Dihydroeugenol isomer		42.190	166	C_10_H_14_O_2_	2116	MS
**M91**	Viridiflorol	552-02-3	42.310	222	C_15_H_26_O	2122	MS, RI
**M92**	Patchoulol	5986-55-0	43.065	222	C_15_H_26_O	2157	MS, RI
**M93**	Eugenol	97-53-0	43.250	164	C_10_H_12_O_2_	2166	MS, RI, REF
**M94**	1,2,4-Trimethoxybenzene isomer		43.565	168	C_9_H_12_O_3_	2181	MS
**M95**	4-Methoxysafrole	18607-93-7	43.745	192	C_11_H_12_O_3_	2189	MS, RI
**M96**	*m*-Eugenol	501-19-9	43.780	164	C_10_H_12_O_2_	2191	MS, RI
**M97**	2,4,5-Trimethoxybenzoic acid	490-64-2	43.815	212	C_10_H_12_O_5_	2192	MS
**M98**	Thymol	89-83-8	43.850	150	C_10_H_14_O	2194	MS, RI
**M99**	*p*-Methoxypropiophenone	121-97-1	43.910	164	C_10_H_12_O_2_	2197	MS
**M100**	Bulnesol	22451-73-6	44.010	222	C_15_H_26_O	2202	MS, RI
**M101**	α-Bisabolol	515-69-5	44.200	222	C_15_H_26_O	2212	MS, RI
**M102**	2-Aminoacetophenone	551-93-9	44.232	135	C_8_H_9_NO	2214	MS, RI
**M103**	α-Eudesmol	473-16-5	44.325	222	C_15_H_26_O	2219	MS, RI
**M104**	Piperonal	120-57-0	44.340	150	C_8_H_6_O_3_	2219	MS, RI
**M105**	Isothymol	499-75-2	44.390	150	C_10_H_14_O	2222	MS, RI
**M106**	α-Cadinol	481-34-5	44.435	222	C_15_H_26_O	2225	MS, RI
**M107**	Elemicin	487-11-6	44.505	208	C_12_H_16_O_3_	2228	MS, RI, REF
**M108**	3,4,5-Trimethoxytoluene isomer		44.765	182	C_10_H_14_O_3_	2242	MS
**M109**	Methoxyeugenol isomer		44.870	194	C_11_H_14_O_3_	2248	MS
**M110**	Isospathulenol	88395-46-4	44.935	220	C_15_H_24_O	2251	MS, RI
**M111**	*cis*-Isoeugenol	5912-86-7	45.080	164	C_10_H_12_O_2_	2258	MS, RI
**M112**	β-Asarone	5273-86-9	45.180	208	C_12_H_16_O_3_	2264	MS, RI, REF
**M113**	1,2,4-Trimethoxybenzene isomer		45.360	168	C_9_H_12_O_3_	2273	MS
**M114**	1,2,3,4-Tetramethoxybenzene	21450-56-6	45.590	198	C_10_H_14_O_4_	2286	MS, RI
**M115**	1,2,4-Trimethoxybenzene isomer		46.360	168	C_9_H_12_O_3_	2334	MS
**M116**	3,4-Methylenedioxyacetophenone	3162-29-6	46.400	164	C_9_H_8_O_3_	2337	MS
**M117**	Chavicol	501-92-8	46.605	134	C_9_H_10_O	2350	MS, RI
**M118**	1,2,4-Trimethoxybenzene isomer		46.670	168	C_9_H_12_O_3_	2355	MS
**M119**	Kaurene	34424-57-2	46.785	272	C_20_H_32_	2362	MS, RI
**M120**	Methylvanillin	120-14-9	47.240	166	C_9_H_10_O_3_	2394	MS, RI
**M121**	Methoxyeugenol isomer		47.304	194	C_11_H_14_O_3_	2398	MS
**M122**	3,4-Methylenedioxypropiophenone	28281-49-4	47.395	178	C_10_H_10_O_3_	2405	MS, REF
**M123**	Apiol	523-80-8	47.510	222	C_12_H_14_O_4_	2415	MS, RI
**M124**	Amilfenol	80-46-6	47.560	164	C_11_H_16_O	2418	MS
**M125**	6-Allyl-2-methylphenol	3354-58-3	47.755	148	C_10_H_12_O	2435	MS
**M126**	Methoxyeugenol isomer		47.835	194	C_11_H_14_O_3_	2441	MS
**M127**	Kakuol isomer		47.895	194	C_10_H_10_O_4_	2446	MS
**M128**	Mellein	1200-93-7	48.255	178	C_10_H_10_O_3_	2475	MS, RI
**M129**	2,4-Dimethoxyacetophenone	829-20-9	48.345	180	C_10_H_12_O_3_	2483	MS
**M130**	2,6-Dimethoxyacetophenone	2040-04-2	48.585	180	C_10_H_12_O_3_	2503	MS
**M131**	1-(3,4-Methylenedioxyphenyl)-propane-1-ol	6890-30-8	48.777	180	C_10_H_12_O_3_	2521	MS
**M132**	Piperonol	495-76-1	48.928	152	C_8_H_8_O_3_	2534	MS
**M133**	Methoxyeugenol isomer		48.935	194	C_11_H_14_O_3_	2534	MS
**M134**	3-Methoxy-5-methylphenol	3209-13-0	48.985	138	C_8_H_10_O_2_	2538	MS, RI
**M135**	2′,4′-Dimethoxypropiophenone	831-00-5	49.045	194	C_11_H_14_O_3_	2544	MS
**M136**	4,6-Dimethoxy-phthalide	58545-97-4	49.065	194	C_10_H_10_O_4_	2546	MS
**M137**	Methoxyeugenol	6627-88-9	49.069	194	C_11_H_14_O_3_	2547	MS, RI
**M138**	Kakuol	18607-90-4	49.295	194	C_10_H_10_O_4_	2567	MS, REF
**M139**	2′,4′-Dimethoxy-3′-methylpropiophenone isomer		49.690	208	C_12_H_16_O_3_	2602	MS
**M140**	Xanthoxylin	90-24-4	49.760	196	C_10_H_10_O_4_	2608	MS
**M141**	3,4-Methylenedioxyphenyl-1-propanal	30830-55-8	49.915	178	C_10_H_10_O_3_	2620	MS
**M142**	2′,4′-Dimethoxy-3′-methylpropiophenone isomer		50.226	208	C_12_H_16_O_3_	2645	MS
**M143**	3,4,5-Trimethoxyphenyl-2-propanone	16603-18-2	50.270	224	C_12_H_16_O_4_	2648	MS
**M144**	4-(Dimethoxymethyl)-1,2-dimethoxybenzene	59276-33-4	50.615	212	C_11_H_16_O_4_	2676	MS
**M145**	Propioveratrone	1835-04-7	50.740	194	C_11_H_14_O_3_	2686	MS
**M146**	2′,4′-Dimethoxypropiophenone	831-00-5	50.760	194	C_11_H_14_O_3_	2687	MS
**M147**	Dihydromethyleugenol	5888-52-8	51.135	180	C_11_H_16_O_2_	2714	MS
**M148**	2′,4′-Dimethoxy-3′-methylpropiophenone	77942-13-3	51.435	208	C_12_H_16_O_3_	2734	MS
**M149**	1-Hydroxy-2-(prop-2-enyl)-4,5- methylenedioxybenzene	19202-23-4	51.670	178	C_10_H_10_O_3_	2750	MS
**M150**	3-(2,4,6-Trimethoxyphenyl)-2-butanone	26537-69-9	51.850	238	C_13_H_18_O_4_	2761	MS
**M151**	1,2-Dimethoxy-4-(1,2-dimethox yethyl)-benzene	477884-01-8	52.195	226	C_12_H_18_O_4_	2785	MS
**M152**	1-Hydroxy-2-(prop-2-enyl)-4,5- methylenedioxybenzene isomer		52.665	178	C_10_H_10_O_3_	2814	MS
**M153**	Syringic acid	530-57-4	55.055	198	C_9_H_10_O_5_	2934	MS

^a^ Compounds identified in feces, urine, blood, and eight organ samples (brain, heart, lung, spleen, liver, kidney, stomach, and small intestine) are sorted by retention time (t_R_), and their polarity increases with prolonged retention time. ^b^ CAS: Chemical Abstracts Service. ^c^ MW: molecular weight. ^d^ RI: calculated based on C_7_–C_30_ saturated alkanes. ^e^ Identification: MS: searching mass spectra in NIST 11 (version 2011, National Institute of Standards and Technology, USA) library embedded in GC–MS workstation (GCMS solutions, version 2.71, Shimadzu, Kyoto, Japan); RI: comparing retention index (RI) and mass spectra with literature; REF: comparing retention time and mass spectra with reference compounds.

**Table 2:** Distribution of 101 original constituents in vivo in rats after oral administration of ARR powder or decoction.

No.	Constituents	RI	Powder-Treated Group											Decoction-Treated Group										
			FE	UR	BL	BR	HE	LU	SP	LI	KI	ST	IN	FE	UR	BL	BR	HE	LU	SP	LI	KI	ST	IN
**M1**	α-Pinene	1021	●																					
**M2**	Camphene	1060	●																					
**M3**	β-Pinene	1099	●									●												
**M4**	Sabinene	1110	●																					
**M5**	3-Carene	1135	●									●	●											
**M6**	β-Myrcene	1154	●									●												
**M7**	*d*-4-Carene	1170										●												
**M8**	Limonene	1191	●									●												
**M9**	Eucalyptol	1203	●							●	●	●	●											
**M10**	*o*-Cymene	1267	●									●											●	
**M11**	Terpinolene	1279	●									●												
**M12**	Tridecane	1293												●										
**M13**	Acetoin	1298												●										
**M14**	*p*-Cymenene	1433										●												
**M16**	*cis*-4-Thujanol	1462	●																					
**M18**	α-Copaene	1485	●																					
**M19**	Pentadecane	1500																					●	
**M20**	*dl*-Camphor	1507	●	●	●		●	●		●	●	●	●	●	●						●	●	●	●
**M21**	1-Pentadecene	1523										●												
**M22**	Eucarvone	1543	●	●	●	●	●	●	●	●	●	●	●									●		
**M23**	*l*-Aristolene	1553	●									●												
**M25**	β-Copaene	1565	●																					
**M26**	Bornyl acetate	1568	●									●		●										
**M27**	1(10)-Aristolene	1577	●									●												
**M28**	Fenchol	1581		●							●													
**M29**	Thymol methyl ether	1590										●	●											
**M30**	*l*-Aristolene isomer	1596										●												
**M31**	β-Cyclocitral	1610		●								●												
**M33**	*l*-Menthol	1621		●																				
**M34**	δ-Guaiene	1626	●									●												
**M35**	Umbellulone	1630		●							●													
**M37**	Estragole	1663			●	●	●	●	●	●	●	●	●											
**M38**	Isomenthol	1670		●																				
**M40**	Eucarvone isomer	1680		●						●	●											●		
**M41**	Verbenone	1684									●													
**M44**	Borneol	1692	●	●	●				●		●	●	●	●	●							●	●	
**M45**	Phellandral	1703		●											●									
**M46**	Piperitone	1706		●						●	●	●												
**M48**	*l*-Carvone	1716		●																				
**M49**	Berbenone	1730		●						●	●	●												
**M50**	*trans*-Piperitol	1745		●																				
**M53**	Myrtenol	1788		●											●									
**M56**	Cuparene	1797										●												
**M57**	*cis*-Sabinol	1799		●																				
**M59**	*cis*-Carveol	1835		●								●			●									
**M60**	3,5-Dimethoxytoluene	1840	●	●	●	●	●	●	●	●	●	●	●								●	●	●	●
**M61**	*p*-Cymen-8-ol	1851		●								●												
**M62**	2,3-Dimethyldecahydronaphthalene	1859									●													
**M64**	Safrole	1862	●	●	●	●	●	●	●	●	●	●	●	●	●					●	●	●		●
**M67**	Verbenone isomer	1895									●													
**M69**	Agarospirol	1928	●																					
**M70**	3,4-Methylenedioxyanisole	1931								●		●												
**M71**	Isosafrole isomer	1936	●																					
**M73**	2-Allyl-1,4-dimethoxybenzene	1968										●												
**M74**	Thymoquinone	1980			●						●				●	●					●	●	●	
**M75**	Methyleugenol isomer	1986										●	●											
**M77**	2,4-Dimethylanisole	2002								●														
**M79**	Methyleugenol	2011	●	●	●	●	●	●	●	●	●	●	●	●	●					●	●	●	●	●
**M81**	Isosafrole	2020	●	●						●	●	●	●	●										
**M82**	2,3,5-Trimethoxytoluene	2040	●	●	●	●	●	●	●	●	●	●	●	●	●	●	●	●	●	●	●	●	●	●
**M83**	3,4,5-Trimethoxytoluene	2048	●	●	●	●	●	●	●	●	●	●	●	●	●	●	●	●	●	●	●	●	●	●
**M84**	3,4,5-Trimethoxybenzoic acid	2055	●		●				●	●	●	●	●									●	●	
**M85**	Globulol	2061	●									●	●											
**M86**	1,2,4-Trimethoxybenzene	2083										●											●	
**M87**	*E*-Isocroweacin	2096	●		●	●	●	●	●	●	●	●	●											
**M89**	Spathulenol	2112	●									●	●											
**M91**	Viridiflorol	2122										●	●											
**M92**	Patchoulol	2157										●	●											
**M93**	Eugenol	2166	●	●				●	●	●	●	●	●	●	●			●	●	●	●	●	●	●
**M95**	4-Methoxysafrole	2189	●		●	●	●		●	●	●	●	●	●									●	
**M97**	2,4,5-Trimethoxybenzoic acid	2192			●					●						●						●	●	
**M98**	Thymol	2194	●	●										●	●									●
**M99**	*p*-Methoxypropiophenone	2197			●	●		●	●		●		●											
**M100**	Bulnesol	2202	●																					
**M101**	α-Bisabolol	2212										●												
**M103**	α-Eudesmol	2219	●																					
**M104**	Piperonal	2219		●	●										●	●								
**M105**	Isothymol	2222										●		●										
**M106**	α-Cadinol	2225	●																					
**M107**	Elemicin	2228			●	●	●			●	●	●	●			●	●			●	●	●	●	●
**M108**	3,4,5-Trimethoxytoluene isomer	2242			●	●				●	●	●	●			●					●	●	●	●
**M109**	Methoxyeugenol isomer	2248		●											●									
**M110**	Isospathulenol	2251										●												
**M112**	β-Asarone	2264			●					●	●	●											●	
**M113**	1,2,4-Trimethoxybenzene isomer	2273	●	●																				
**M114**	1,2,3,4-Tetramethoxybenzene	2286	●	●											●									
**M116**	3,4-Methylenedioxyacetophenone	2337		●	●										●	●							●	
**M117**	Chavicol	2350		●							●				●						●	●	●	●
**M119**	Kaurene	2362	●									●												
**M122**	3,4-Methylenedioxypropiophenone	2405	●	●	●	●	●	●	●	●	●	●	●	●	●	●	●	●	●	●	●	●	●	●
**M123**	Apiol	2415										●												
**M126**	Methoxyeugenol isomer	2441	●	●											●								●	
**M127**	Kakuol isomer	2446	●	●							●	●	●	●	●							●	●	
**M128**	Mellein	2475		●								●	●		●								●	
**M131**	1-(3,4-Methylenedioxyphenyl)-propane-1-ol	2520	●		●				●	●	●		●	●	●						●	●	●	●
**M135**	2′,4′-Dimethoxypropiophenone	2544	●	●																				
**M138**	Kakuol	2567	●	●	●	●	●	●	●	●	●	●	●	●	●	●	●	●	●	●	●	●	●	●
**M139**	2′,4′-Dimethoxy-3′-methylpropiophenone isomer	2602									●	●	●		●							●	●	●
**M140**	Xanthoxylin	2608	●	●							●	●	●		●								●	●
**M148**	2′,4′-Dimethoxy-3′-methylpropiophenone	2734	●		●	●	●	●	●	●	●	●	●	●	●	●			●	●	●	●	●	●
**M153**	Syringic acid	2934		●																				
Total:			49	40	24	15	14	14	17	27	36	59	33	19	26	11	5	5	6	9	15	22	28	17
			Total 98 original constituents were identified in the powder group.											Total 43 original constituents were identified in the decoction group.										

RI: calculating based on the C_7_–C_30_ saturated alkanes; FE: feces; UR: urine; BL: blood; BR: brain; HE: heart; LU: lung; SP: spleen; LI: liver; KI: kidney; ST: stomach; IN: small intestine; ●: detected.

**Table 3:** Distribution of 15 metabolites in vivo in rats after oral administration of ARR powder or decoction.

No.	Metabolites	RI	Powder-Treated Group											Decoction-Treated Group										
			FE	UR	BL	BR	HE	LU	SP	LI	KI	ST	IN	FE	UR	BL	BR	HE	LU	SP	LI	KI	ST	IN
**M15**	*cis*-Limonene oxide	1441		●							●											●		
**M24**	Isopulegol	1556	●	●																				
**M36**	*l*-Pinocarveol	1651		●											●									
**M39**	Verbenol	1674									●													
**M42**	α-Terpineol acetate	1685										●												
**M47**	β-Cyclogeraniol	1711													●									
**M51**	*cis*-Piperitol	1750		●																				
**M88**	Dihydroeugenol	2106		●						●	●		●		●						●			
**M96**	*m*-Eugenol	2191		●																				
**M111**	*cis*-Isoeugenol	2258		●																				
**M132**	Piperonol	2534													●									
**M137**	Methoxyeugenol	2547													●									
**M141**	3,4-Methylenedioxyphenyl-1-propanal	2620		●																				
**M147**	Dihydromethyleugenol	2714									●													
**M149**	1-Hydroxy-2-(prop-2-enyl)-4,5-methylenedioxybenzene	2750		●																				
Total:			1	9	0	0	0	0	0	1	4	1	1	0	5	0	0	0	0	0	1	1	0	0
			Total 12 metabolites were identified in the powder group.											Total 6 metabolites were identified in the decoction group.										

RI: calculating based on the C_7_–C_30_ saturated alkanes; FE: feces; UR: urine; BL: blood; BR: brain; HE: heart; LU: lung; SP: spleen; LI: liver; KI: kidney; ST: stomach; IN: small intestine. ●: detected.

**Table 4:** Peak areas of main absorbed constituents and their ratio between different administration groups (×10^6^).

	3,5-Dimethoxytoluene (M60)			Safrole (M64)			Methyleugenol (M79)			2,3,5-Trimethoxytoluene (M82)			3,4,5-Trimethoxytoluene (M83)			Eugenol (M93)			Kakuol (M138)		
	P	D	Ratio	P	D	Ratio	P	D	Ratio	P	D	Ratio	P	D	Ratio	P	D	Ratio	P	D	Ratio
FE	7.19	n.d.	/	3.25	0.0058	560.3	2.04	0.0052	392.3	3.45	0.005	690.0	4.07	0.01	407.0	0.10	0.02	5.4	1.36	0.70	1.9
UR	0.54	n.d.	/	0.04	0.0038	10.5	0.12	0.0064	18.8	0.65	0.030	21.7	1.10	0.32	3.4	5.66	6.58	0.9	16.79	10.51	1.6
BL	2.68	n.d.	/	2.54	n.d.	/	0.5	n.d.	/	1.91	0.032	59.7	2.11	0.31	6.8	n.d.	n.d.	/	0.10	0.02	6.4
BR	2.48	n.d.	/	1.94	n.d.	/	0.4	n.d.	/	0.75	0.007	107.1	0.64	0.06	10.7	n.d.	n.d.	/	0.07	0.01	13.0
HE	1.45	n.d.	/	0.98	n.d.	/	0.26	n.d.	/	0.54	0.007	77.1	0.46	0.05	9.2	n.d.	0.004	/	0.06	0.005	12.5
LU	1.27	n.d.	/	0.98	n.d.	/	0.24	n.d.	/	0.39	0.008	48.8	0.33	0.05	6.6	0.05	0.01	5.2	0.09	0.01	10.9
SP	1.86	n.d.	/	1.43	0.0004	3575.0	0.9	0.0016	562.5	0.97	0.009	107.8	0.59	0.06	9.8	0.02	0.01	3.0	0.16	0.01	21.7
LI	7.09	0.0017	4170.6	5.03	0.0012	4191.7	1.29	0.0032	403.1	2.26	0.031	72.9	1.89	0.24	7.9	0.05	0.02	2.6	0.05	0.04	1.2
KI	4.63	0.0019	2436.8	2.48	0.0018	1377.8	0.88	0.0051	172.5	2.58	0.056	46.1	2.78	0.56	5.0	0.22	0.23	0.9	1.13	0.31	3.6
ST	35.16	0.0025	14,064.0	46.54	n.d.	/	34.99	0.0801	436.8	18.84	0.293	64.3	17.07	1.90	9.0	0.20	0.05	4.1	8.58	2.36	3.6
IN	13.06	0.0021	6219.0	16.02	0.0025	6408.0	12.38	0.0069	1794.2	6.56	0.026	252.3	5.83	0.13	44.8	0.06	0.12	0.5	2.01	0.22	9.2

P: powder-treated group; D: decoction-treated group; FE: feces; UR: urine; BL: blood; BR: brain; HE: heart; LU: lung; SP: spleen; LI: liver; KI: kidney; ST: stomach; IN: small intestine. Ratio: the ratio of peak areas between powder- and decoction-treated groups. n.d.: not detected. /: The ratio cannot be calculated because the constituents failed to be detected in the powder- or decoction-treated group.

**Table 5:** Acute toxicity and distribution of the compounds identified in the blood of powder-treated group.

No.	Compounds	LD_50_ (Oral) ^a^	Distribution ^b^	Content ^c^
**M20**	*dl*-Camphor	mouse, 1310 mg/kg [35]	P (FE, UR, BL, HE, LU, LI, KI, ST, IN) D (FE, UR, LI, KI, ST, IN)	0.11%
**M37**	Estragole	mouse, 1250 mg/kg [36]	P (BL, BR, HE, LU, SP, LI, KI, ST, IN) D (n.d.)	0.28%
**M44**	Borneol	mouse, 1059 mg/kg [35]	P (FE, UR, BL, SP, KI, ST, IN) D (FE, UR, KI, ST)	0.12%
**M64**	safrole	mouse, 2350 mg/kg [33,36]	P (FE, UR, BL, BR, HE, LU, SP, LI, KI, ST, IN) D (FE, UR, SP, LI, KI, IN)	23.74%
**M79**	Methyleugenol	rat, 1179 mg/kg [33]	P (FE, UR, BL, BR, HE, LU, SP, LI, KI, ST, IN) D (FE, UR, SP, LI, KI, ST, IN)	4.69%
**M104**	Piperonal	rat, 2700 mg/kg [37]	P (UR, BL) D (UR, BL)	0.07%
**M112**	β-Asarone	mouse, 418 mg/kg [38]	P (BL, LI, KI, ST) D (ST)	0.04%
**M122**	3,4-Methylenedioxypropiophenone	mouse, 2150 mg/kg [39]	P (FE, UR, BL, BR, HE, LU, SP, LI, KI, ST, IN) D (FE, UR, BL, BR, HE, LU, SP, LI, KI, ST, IN)	1.58%
**M131**	1-(3,4-Methylenedioxyphenyl)-propane-1-ol	mouse, 720 mg/kg [40]	P (FE, BL, SP, LI, KI, IN) D (FE, UR, LI, KI, ST, IN)	0.15%

^a^ The LD_50_ values were queried in PubChem (https://pubchem.ncbi.nlm.nih.gov/). ^b^ Distribution in this study, P: powder-treated group; D: decoction-treated group; FE: feces; UR: urine; BL: blood; BR: brain; HE: heart; LU: lung; SP: spleen; LI: liver; KI: kidney; ST: stomach; IN: small intestine. ^c^ The relative content in the blood of powder-treated group.

## Supplementary Materials

The following are available online: Figures S1–S2, Tables S1–S23.

## Abbreviations

ARR	Asari Radix et Rhizoma
AHM	*Asarum heterotropoides* Fr. Schmidt var. *mandshuricum* (Maxim.) Kitag.
VOCs	Volatile organic compounds
RI	Retention index
LD_50_	Median lethal dose
3,5-DMT	3,5-Dimethoxytoluene
2,3,5-TMT	2,3,5-Trimethoxytoluene
3,4,5-TMT	3,4,5-Trimethoxytoluene
GC–MS	Gas chromatography–mass spectrometry
HPLC	High–performance liquid chromatography
UPLC	Ultra–performance liquid chromatography
HS–SPME–GC–MS	Headspace solid–phase microextraction gas–chromatographymass spectrometry
HPLC–APCI–IT–TOF–MS^n^	High-performance liquid chromatography–atmospheric pressurechemical ionization–ion trap–time of flight–multistage mass spectrometry
