# Supplementary material for: The Relative Content and Distribution of Absorbed Volatile Organic Compounds in Rats Administered Asari Radix et Rhizoma Are Different between Powder- and Decoction-Treated Groups
Source: Molecules. 2020 Sep 27;25(19):4441. doi: 10.3390/molecules25194441 (PMC7582631; doi:10.3390/molecules25194441)
Supplement: Supplementary file 1 [file molecules-25-04441-s001.pdf]

The relative content and distribution of absorbed volatile organic compounds in rats administered Asari Radix et Rhizoma are different between powder- and decoction-treated groups

Guang-Xue Liu[1], Feng Xu[1], Ming-Ying Shang[1,*], Xuan Wang[2] and Shao-Qing Cai[1,*]

Supplement materials

Figures

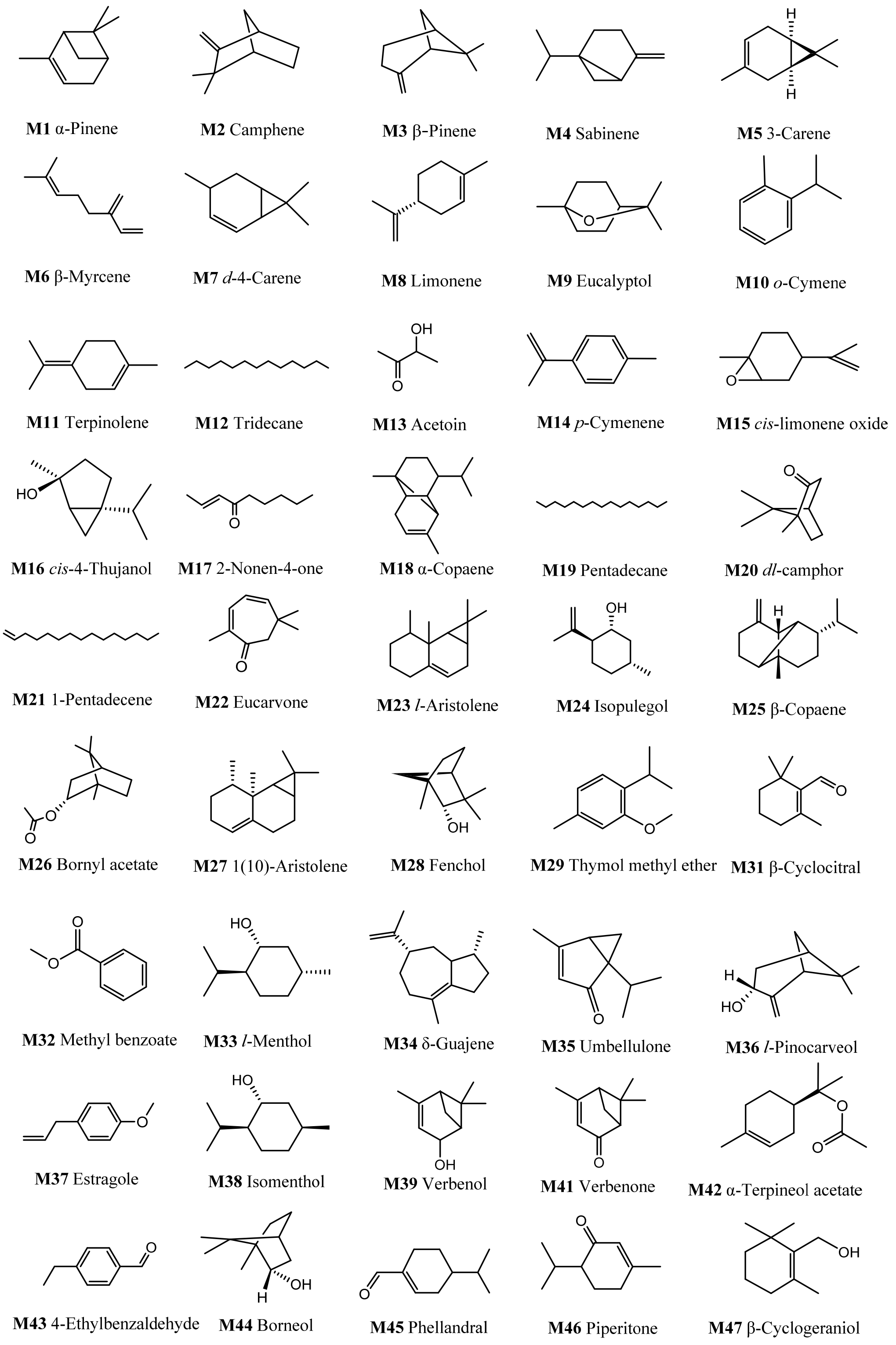

Figure S1-1. The volatile compounds identified *in vivo* in rats.

As some compounds can only be identified as isomers, the structures of them are not shown in the figure.

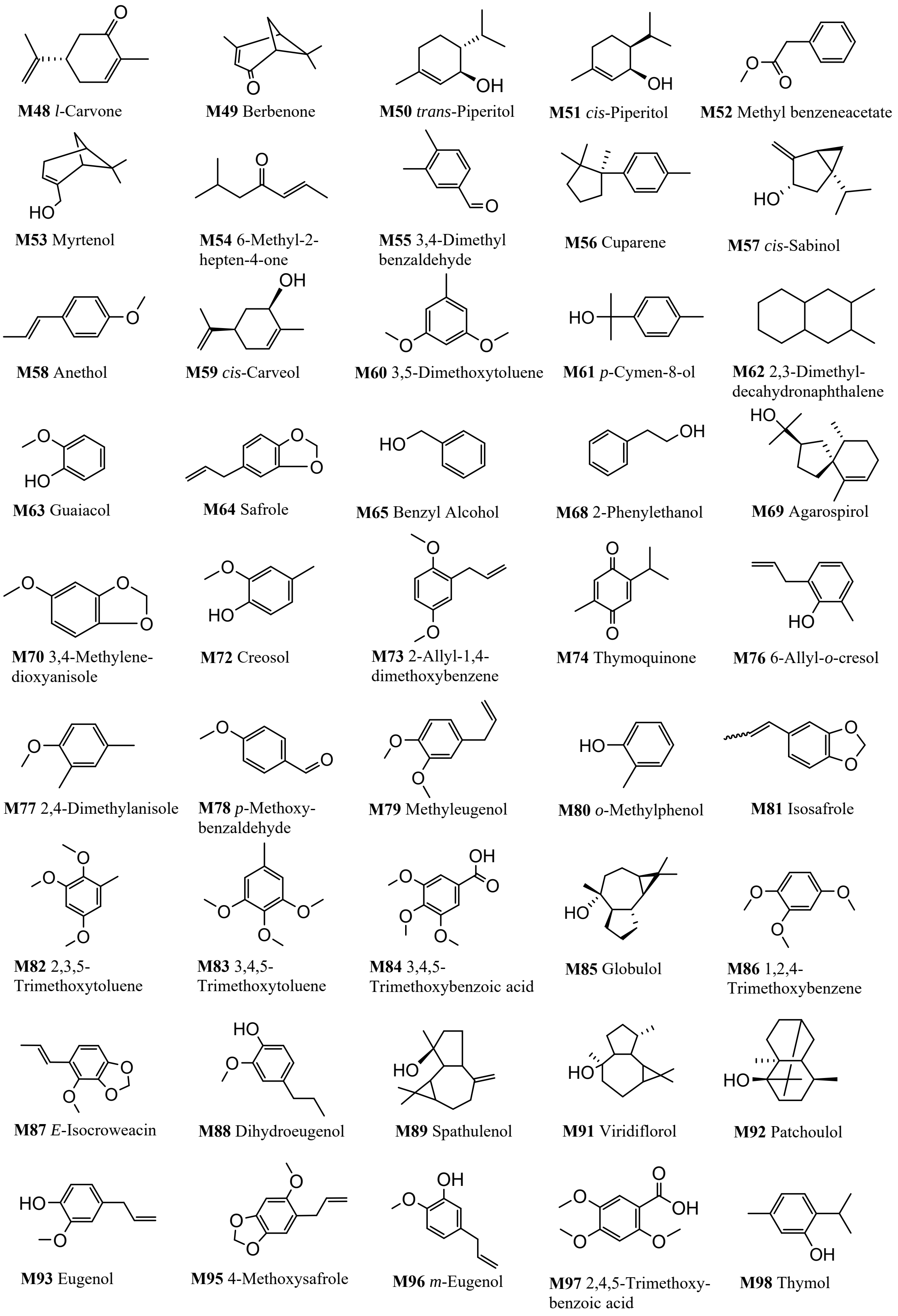

Figure S1-2. The volatile compounds identified *in vivo* in rats.

As some compounds can only be identified as isomers, the structures of them are not shown in the figure.

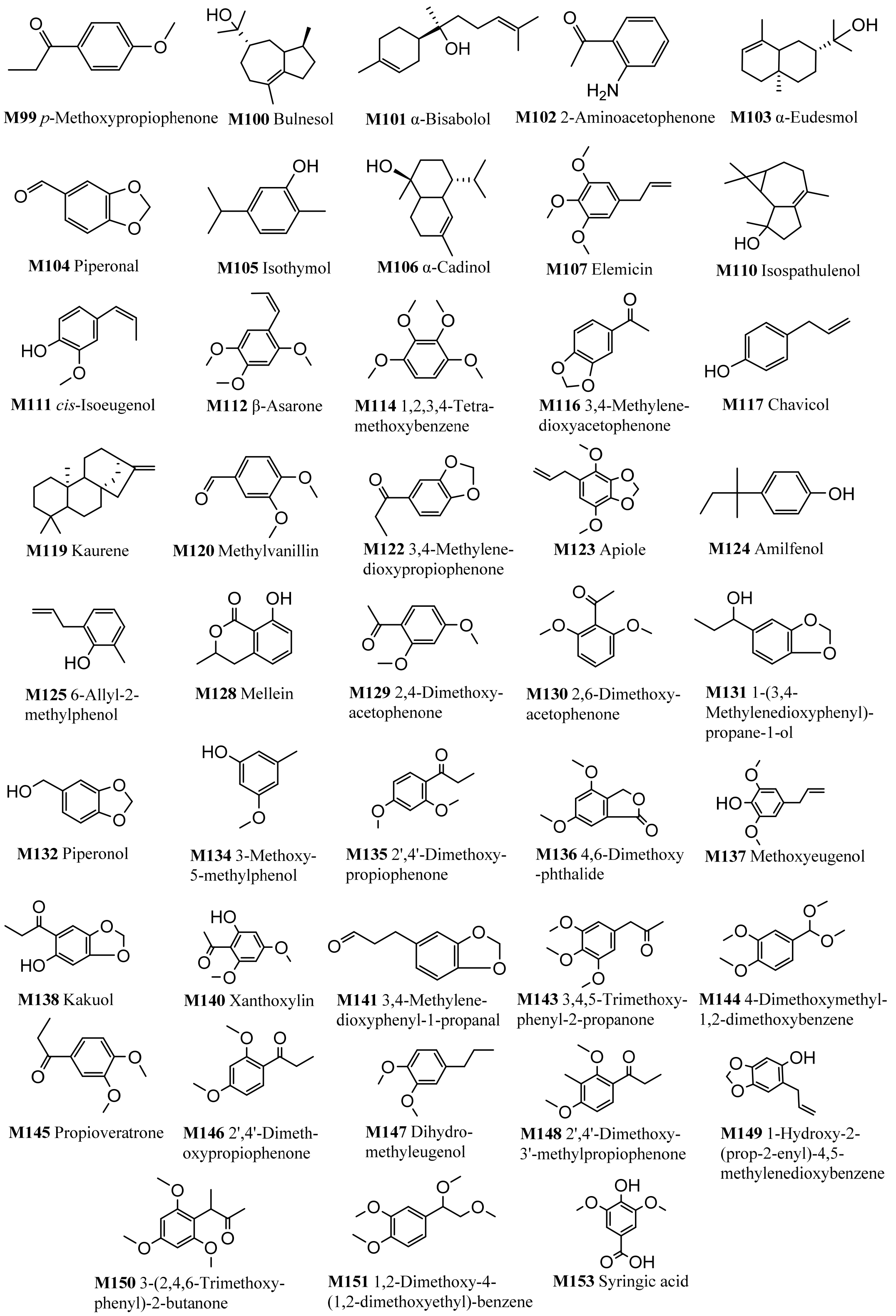

Figure S1-3. The volatile compounds identified *in vivo* in rats.

As some compounds could only be identified as isomers, the structures of them were not shown in the figure.

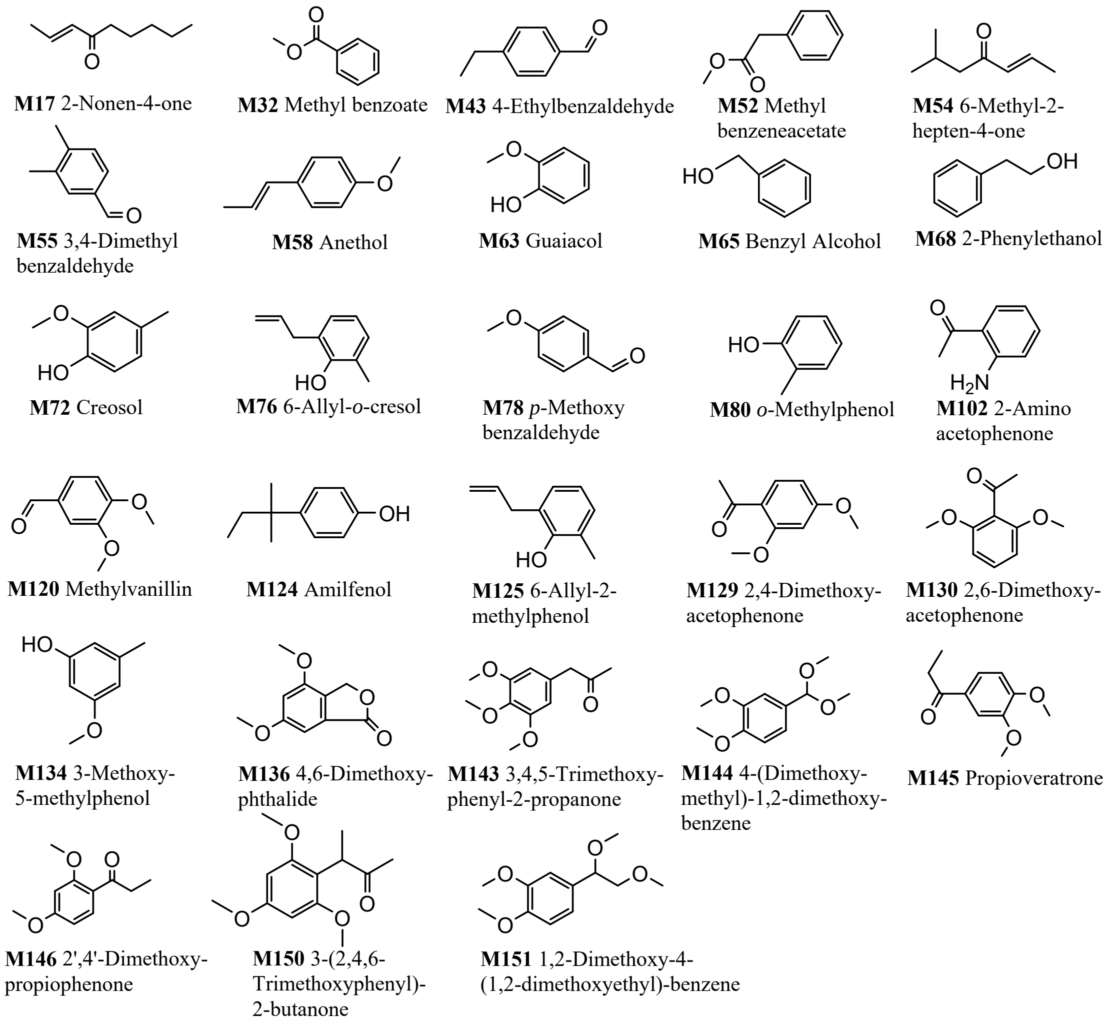

Figure S2. The unclassified compounds identified *in vivo* in rats.

Unclassified compounds were identified in the ARR treated groups, but could not be found in either the control group or the ARR sample itself, suggesting that they might be metabolites of constituents or endogenous substances generated after the administration of ARR. As some compounds could only be identified as isomers, the structures of them were not shown in the figure.

Tables

Table S1. Distribution of 37 unclassified compounds *in vivo* in rats after oral administration of ARR powder or decoction

No.	Unclassified Compounds	RI	Powder-treated group											Decoction-treated group										
			FE	UR	BL	BR	HE	LU	SP	LI	KI	ST	IN	FE	UR	BL	BR	HE	LU	SP	LI	KI	ST	IN
**M17**	2-Nonen-4-one	1477		●																				
**M32**	Methyl benzoate	1615													●									
**M43**	4-Ethylbenzaldehyde	1693				●	●														●			
**M52**	Methyl benzeneacetate	1758		●																				
**M54**	6-Methyl-2-hepten-4-one	1790	●																					
**M55**	3,4-Dimethylbenzaldehyde	1790													●									
**M58**	Anethol	1815	●																					
**M63**	Guaiacol	1860													●									
**M65**	Benzyl alcohol	1877													●									
**M66**	Verbenone isomer	1891																				●		
**M68**	2-Phenylethanol	1906													●									
**M72**	Creosol	1954													●									
**M76**	6-Allyl-*o*-cresol	2000													●									
**M78**	*p*-Methoxybenzaldehyde	2008		●																				
**M80**	*o*-Methylphenol	2012		●																				
**M90**	Dihydroeugenol isomer	2116		●											●									
**M94**	1,2,4-Trimethoxybenzene isomer	2181		●							●				●									
**M102**	2-Aminoacetophenone	2214												●										
**M115**	1,2,4-Trimethoxybenzene isomer	2334		●				●			●				●				●		●	●		●
**M118**	1,2,4-Trimethoxybenzene isomer	2355		●											●									
**M120**	Methylvanillin	2394		●																				
**M121**	Methoxyeugenol isomer	2398		●											●									
**M124**	Amilfenol	2418		●																				
**M125**	6-Allyl-2-methylphenol	2435		●																				
**M129**	2,4-Dimethoxyacetophenone	2483		●																				
**M130**	2,6-Dimethoxyacetophenone	2503													●									
**M133**	Methoxyeugenol isomer	2534		●																				
**M134**	3-Methoxy-5-methylphenol	2538		●											●									●

No.	Unclassified Compounds	RI	Powder-treated group											Decoction-treated group										
			FE	UR	BL	BR	HE	LU	SP	LI	KI	ST	IN	FE	UR	BL	BR	HE	LU	SP	LI	KI	ST	IN
**M136**	4,6-Dimethoxy-phthalide	2546																					●	
**M142**	2',4'-Dimethoxy-3'-methylpropiophenone isomer	2645																				●		
**M143**	3,4,5-Trimethoxyphenyl-2-propanone	2648		●											●									
**M144**	4-(Dimethoxymethyl)-1,2-dimethoxybenzene	2676										●											●	
**M145**	Propioveratrone	2686		●																			●	
**M146**	2',4'-Dimethoxypropiophenone	2687													●									
**M150**	3-(2,4,6-Trimethoxyphenyl)-2-butanone	2761		●																				
**M151**	1,2-Dimethoxy-4-(1,2-dimethoxyethyl)-benzene	2785										●											●	
**M152**	1-Hydroxy-2-(prop-2-enyl)-4,5-methylenedioxybenzene isomer	2814		●											●									
		Total:	2	19	0	1	1	1	0	0	2	2	0	1	17	0	0	0	1	0	2	3	4	2
			Total 24 unclassified compounds were identified in the powder group.											Total 25 unclassified compounds were identified in the decoction group.										

RI: calculating based on the C_7-C_{30} saturated alkanes; FE: feces; UR: urine; BL: blood; BR: Brain; HE: heart; LU: lung; SP: spleen; LI: liver; KI: kidney; ST: stomach; IN: small intestine; ●: detected.

Unclassified compounds were identified in the ARR treated groups, but could not be found in either the control group or the ARR sample itself.

Table S2. The information of 52 VOCs identified in the feces of rats in ARR powder-treated group by HS-SPME-GC-MS

No. [a]	Compounds	t_R (min)	MW	Formula	Ion [b]	Area	C (%) [c]	Identification [d]	RI [e]
**M1**	α-Pinene	3.785	136	$C_{10}H_{16}$	93	171932	0.49	MS, RI	1021
**M2**	Camphene	4.375	136	$C_{10}H_{16}$	93	32827	0.09	MS, RI	1060
**M3**	β-Pinene	4.955	136	$C_{10}H_{16}$	93	245739	0.70	MS, RI, REF	1099
**M4**	Sabinene	5.175	136	$C_{10}H_{16}$	93	31865	0.09	MS, RI	1110
**M5**	3-Carene	5.700	136	$C_{10}H_{16}$	93	66866	0.19	MS, RI	1135
**M6**	β-Myrcene	6.080	136	$C_{10}H_{16}$	93	23037	0.07	MS, RI	1154
**M8**	Limonene	6.830	136	$C_{10}H_{16}$	93	22517	0.06	MS, RI, REF	1191
**M9**	Eucalyptol	7.095	154	$C_{10}H_{18}O$	81	62464	0.18	MS, RI	1204
**M10**	*o*-Cymene	8.630	134	$C_{10}H_{14}$	119	70263	0.20	MS, RI	1267
**M11**	Terpinolene	8.895	136	$C_{10}H_{16}$	93	8324	0.02	MS, RI	1279
**M16**	*cis*-4-Thujanol	13.795	154	$C_{10}H_{18}O$	111	4598	0.01	MS, RI	1462
**M18**	α-Copaene	14.485	204	$C_{15}H_{24}$	161	16753	0.05	MS, RI	1485
**M20**	*dl*-Camphor	15.305	152	$C_{10}H_{16}O$	95	87854	0.25	MS, RI	1507
**M22**	Eucarvone	17.060	150	$C_{10}H_{14}O$	107	141683	0.40	MS, REF	1543
**M23**	*l*-Aristolene	17.560	204	$C_{15}H_{24}$	105	28432	0.08	MS, RI	1553
**M24**	Isopulegol	17.720	154	$C_{10}H_{18}O$	139	3377	0.01	MS, RI	1556
**M25**	β-Copaene	18.115	204	$C_{15}H_{24}$	161	12845	0.04	MS, RI	1565
**M26**	Bornyl acetate	18.305	196	$C_{12}H_{20}O_2$	95	33587	0.10	MS, RI	1568
**M27**	1(10)-Aristolene	18.760	204	$C_{15}H_{24}$	161	190475	0.54	MS, RI	1577
**M34**	δ-Guaiene	21.535	204	$C_{15}H_{24}$	93	52101	0.15	MS, RI	1626
**M44**	Borneol	25.875	154	$C_{10}H_{18}O$	95	4673426	13.32	MS, RI	1694
**M54**	6-Methyl-2-hepten-4-one	32.060	126	$C_8H_{14}O$	111	24937	0.07	MS	1790
**M58**	Anethol	33.265	148	$C_{10}H_{12}O$	148	56098	0.16	MS, RI	1815
**M60**	3,5-Dimethoxytoluene	34.260	152	$C_9H_{12}O_2$	152	7192478	20.51	MS, RI, REF	1841
**M64**	Safrole	35.110	162	$C_{10}H_{10}O_2$	162	3252027	9.27	MS, RI, REF	1863
**M69**	Agarospirol	37.365	222	$C_{15}H_{26}O$	207	33421	0.10	MS	1928
**M71**	Isosafrole isomer	37.575	162	$C_{10}H_{10}O_2$	162	52447	0.15	MS	1936
**M79**	Methyleugenol	39.715	178	$C_{11}H_{14}O_2$	178	2044322	5.83	MS, RI, REF	2011
**M81**	Isosafrole	39.925	162	$C_{10}H_{10}O_2$	162	2345048	6.69	MS, RI	2020
**M82**	2,3,5-Trimethoxytoluene	40.415	182	$C_{10}H_{14}O_3$	167	3452503	9.84	REF	2040
**M83**	3,4,5-Trimethoxytoluene	40.610	182	$C_{10}H_{14}O_3$	182	4069981	11.60	MS, RI, REF	2048
**M84**	3,4,5-Trimethoxybenzoic acid	40.780	212	$C_{10}H_{12}O_5$	212	40435	0.12	MS	2056
**M85**	Globulol	40.910	222	$C_{15}H_{26}O$	95	21896	0.06	MS, RI	2061
**M87**	*E*-Isocroweacin	41.755	192	$C_{11}H_{12}O_3$	192	541442	1.54	MS	2096
**M89**	Spathulenol	42.110	220	$C_{15}H_{24}O$	205	17215	0.05	MS, RI	2112
**M93**	Eugenol	43.250	164	$C_{10}H_{12}O_2$	164	101999	0.29	MS, RI, REF	2166
**M95**	4-Methoxysafrole	43.745	192	$C_{11}H_{12}O_3$	192	2347538	6.69	MS, RI	2189
**M98**	Thymol	43.850	150	$C_{10}H_{14}O$	135	594176	1.69	MS, RI	2194
**M100**	Bulnesol	44.010	222	$C_{15}H_{26}O$	161	20620	0.06	MS, RI	2202
**M103**	α-Eudesmol	44.325	222	$C_{15}H_{26}O$	149	26197	0.07	MS, RI	2219
**M106**	α-Cadinol	44.435	222	$C_{15}H_{26}O$	121	37560	0.11	MS, RI	2225
**M113**	1,2,4-Trimethoxybenzene isomer	45.360	168	$C_9H_{12}O_3$	168	556981	1.59	MS	2273
**M114**	1,2,3,4-Tetramethoxybenzene	45.590	198	$C_{10}H_{14}O_4$	198	123993	0.35	MS, RI	2286
**M119**	Kaurene	46.785	272	$C_{20}H_{32}$	257	14646	0.04	MS, RI	2362
**M122**	3,4-Methylenedioxypropiophenone	47.395	178	$C_{10}H_{10}O_3$	149	400045	1.14	MS, REF	2405
**M126**	Methoxyeugenol isomer	47.835	194	$C_{11}H_{14}O_3$	194	15324	0.04	MS	2441
**M127**	Kakuol isomer	47.895	194	$C_{10}H_{10}O_4$	165	116814	0.33	MS	2447
**M131**	1-(3,4-Methylenedioxyphenyl)-propane-1-ol	48.770	180	$C_{10}H_{12}O_3$	151	13032	0.04	MS	2520
**M135**	2',4'-Dimethoxypropiophenone	49.045	194	$C_{11}H_{14}O_3$	165	22552	0.06	MS	2544
**M138**	Kakuol	49.295	194	$C_{10}H_{10}O_4$	165	1360464	3.88	MS, REF	2567
**M140**	Xanthoxylin	49.760	196	$C_{10}H_{10}O_4$	181	42216	0.12	MS	2608
**M148**	2',4'-Dimethoxy-3'-methylpropiophenone	51.435	208	$C_{12}H_{16}O_3$	179	155025	0.44	MS	2734

[a] Compounds identified in feces, urine, blood, and eight organ samples (brain, heart, lung, spleen, liver, kidney, stomach, and small intestine) are sorted by retention time (t_R), and their polarity increases with prolonged retention time. [b] Ion and [c] C (%): Contents were calculated from extracted ion chromatography (EIC) according to area generalization method. [d] Identification: MS: searching mass spectra in NIST 11(version 2011, National Institute of Standards and Technology, USA) library embedded in GC-MS workstation (GCMS solutions, version 2.71, Shimadzu, Kyoto, Japan); RI: comparing retention index (RI) and mass spectra with literature; REF: comparing retention time and mass spectra with reference compounds. [e] RI: calculating based on the C_7–C_{30} saturated alkanes.
The note of Table S2 is also suitable for Tables S3–S23 in the supplement material.

Table S3. The information of 68 VOCs identified in the urine of rats in ARR powder-treated group by HS-SPME-GC-MS

No.	Compounds	t_R (min)	MW	Formula	Ion	Area	C (%)	Identification	RI
**M15**	*cis*-Limonene oxide	13.135	152	$C_{10}H_{16}O$	137	4428	0.01	MS, RI	1441
**M17**	2-Nonen-4-one	14.270	140	$C_9H_{16}O$	69	57292	0.12	MS, RI	1477
**M20**	*dl*-Camphor	15.340	152	$C_{10}H_{16}O$	95	128740	0.27	MS, RI	1508
**M22**	Eucarvone	17.095	150	$C_{10}H_{14}O$	107	821759	1.75	MS, REF	1544
**M24**	Isopulegol	17.710	154	$C_{10}H_{18}O$	139	3632	0.01	MS, RI	1556
**M28**	Fenchol	18.920	154	$C_{10}H_{18}O$	81	18757	0.04	MS, RI	1581
**M31**	β-Cyclocitral	20.510	152	$C_{10}H_{16}O$	137	12398	0.03	MS, RI	1610
**M33**	*l*-Menthol	21.220	156	$C_{10}H_{20}O$	95	10123	0.02	MS, RI	1621
**M35**	Umbellulone	21.815	150	$C_{10}H_{14}O$	108	22890	0.05	MS, RI	1630
**M36**	*l*-Pinocarveol	23.120	152	$C_{10}H_{16}O$	92	33448	0.07	MS, RI	1651
**M38**	Isomenthol	24.295	156	$C_{10}H_{20}O$	59	10354	0.02	MS, RI	1670
**M40**	Eucarvone isomer	24.990	150	$C_{10}H_{14}O$	107	65723	0.14	MS	1680
**M44**	Borneol	25.895	154	$C_{10}H_{18}O$	95	4981465	10.58	MS, RI	1695
**M45**	Phellandral	26.415	154	$C_{10}H_{18}O$	109	34705	0.07	MS, RI	1703
**M46**	Piperitone	26.610	152	$C_{10}H_{16}O$	82	22856	0.05	MS, RI	1706
**M47**	β-Cyclogeraniol	26.960	154	$C_{10}H_{18}O$	93	5992	0.01	MS	1711
**M48**	*l*-Carvone	27.270	150	$C_{10}H_{14}O$	82	60143	0.13	MS, RI	1716
**M49**	Berbenone	28.190	150	$C_{10}H_{14}O$	150	34403	0.07	MS, RI	1730
**M50**	*trans*-Piperitol	29.165	154	$C_{10}H_{18}O$	84	10429	0.02	MS, RI	1745
**M51**	*cis*-Piperitol	29.485	154	$C_{10}H_{18}O$	84	28096	0.06	MS, RI	1750
**M52**	Methyl benzeneacetate	29.955	150	$C_9H_{10}O_2$	91	28669	0.06	MS, RI	1758
**M53**	Myrtenol	31.910	152	$C_{10}H_{16}O$	108	5924	0.01	MS, RI	1788
**M57**	*cis*-Sabinol	32.645	152	$C_{10}H_{16}O$	92	70666	0.15	MS, RI	1799
**M59**	*cis*-Carveol	34.050	152	$C_{10}H_{16}O$	109	22829	0.05	MS, RI	1835
**M60**	3,5-Dimethoxytoluene	34.265	152	$C_9H_{12}O_2$	152	544677	1.16	MS, RI, REF	1841
**M61**	*p*-Cymen-8-ol	34.660	150	$C_{10}H_{14}O$	135	261325	0.55	MS, RI	1851
**M64**	Safrole	35.130	162	$C_{10}H_{10}O_2$	162	44345	0.09	MS, RI, REF	1863
**M78**	*p*-Methoxybenzaldehyde	39.630	136	$C_8H_8O_2$	135	35202	0.07	MS, RI	2008
**M79**	Methyleugenol	39.710	178	$C_{11}H_{14}O_2$	178	124210	0.26	MS, RI, REF	2011
**M80**	*o*-Methylphenol	39.730	108	C_7H_8O	108	173080	0.37	MS, RI	2012
**M81**	Isosafrole	39.925	162	$C_{10}H_{10}O_2$	162	8693	0.02	MS, RI	2020
**M82**	2,3,5-Trimethoxytoluene	40.415	182	$C_{10}H_{14}O_3$	167	653061	1.39	REF	2040
**M83**	3,4,5-Trimethoxytoluene	40.610	182	$C_{10}H_{14}O_3$	182	1098531	2.33	MS, RI, REF	2048
**M88**	Dihydroeugenol	41.970	166	$C_{10}H_{14}O_2$	137	4465141	9.48	MS, RI	2106
**M90**	Dihydroeugenol isomer	42.190	166	$C_{10}H_{14}O_2$	137	344271	0.73	MS	2116
**M93**	Eugenol	43.255	164	$C_{10}H_{12}O_2$	164	5663865	12.03	MS, RI, REF	2166
**M94**	1,2,4-Trimethoxybenzene isomer	43.565	168	$C_9H_{12}O_3$	153	1270131	2.70	MS	2181
**M96**	*m*-Eugenol	43.780	164	$C_{10}H_{12}O_2$	164	266429	0.57	MS, RI	2191
**M98**	Thymol	43.850	150	$C_{10}H_{14}O$	135	302999	0.64	MS, RI	2194
**M104**	Piperonal	44.340	150	$C_8H_6O_3$	149	798155	1.70	MS, RI	2219
**M109**	Methoxyeugenol isomer	44.870	194	$C_{11}H_{14}O_3$	194	28455	0.06	MS	2248
**M111**	*cis*-Isoeugenol	45.080	164	$C_{10}H_{12}O_2$	164	43456	0.09	MS, RI	2258
**M113**	1,2,4-Trimethoxybenzene isomer	45.325	168	$C_9H_{12}O_3$	168	487523	1.04	MS	2273
**M114**	1,2,3,4-Tetramethoxybenzene	45.595	198	$C_{10}H_{14}O_4$	198	44397	0.09	MS, RI	2286
**M115**	1,2,4-Trimethoxybenzene isomer	46.360	168	$C_9H_{12}O_3$	153	258162	0.55	MS	2334
**M116**	3,4-Methylenedioxyacetophenone	46.400	164	$C_9H_8O_3$	149	241710	0.51	MS	2337
**M117**	Chavicol	46.605	134	$C_9H_{10}O$	134	510964	1.09	MS, RI	2350
**M118**	1,2,4-Trimethoxybenzene isomer	46.670	168	$C_9H_{12}O_3$	168	161265	0.34	MS	2355
**M120**	Methylvanillin	47.240	166	$C_9H_{10}O_3$	166	54498	0.12	MS, RI	2394
**M122**	3,4-Methylenedioxypropiophenone	47.400	178	$C_{10}H_{10}O_3$	149	456447	0.97	MS, REF	2405
**M124**	Amilfenol	47.560	164	$C_{11}H_{16}O$	135	6738	0.01	MS	2418
**M125**	6-Allyl-2-methylphenol	47.755	148	$C_{10}H_{12}O$	148	10119	0.02	MS	2435
**M126**	Methoxyeugenol isomer	47.830	194	$C_{11}H_{14}O_3$	194	287677	0.61	MS	2441
**M127**	Kakuol isomer	47.900	194	$C_{10}H_{10}O_4$	165	3467158	7.36	MS	2447
**M128**	Mellein	48.255	178	$C_{10}H_{10}O_3$	178	36932	0.08	MS, RI	2475
**M129**	2,4-Dimethoxyacetophenone	48.345	180	$C_{10}H_{12}O_3$	165	50946	0.11	MS	2483
**M133**	Methoxyeugenol isomer	48.935	194	$C_{11}H_{14}O_3$	194	73982	0.16	MS	2534
**M134**	3-Methoxy-5-methylphenol	48.985	138	$C_8H_{10}O_2$	138	573184	1.22	MS, RI	2538
**M135**	2',4'-Dimethoxypropiophenone	49.050	194	$C_{11}H_{14}O_3$	165	79181	0.17	MS	2544
**M138**	Kakuol	49.305	194	$C_{10}H_{10}O_4$	165	16790389	35.66	MS, REF	2568
**M140**	Xanthoxylin	49.755	196	$C_{10}H_{10}O_4$	181	482144	1.02	MS	2608
**M141**	3,4-Methylenedioxyphenyl-1-propanal	49.915	178	$C_{10}H_{10}O_3$	178	72502	0.15	MS	2620
**M143**	3,4,5-Trimethoxyphenyl-2-propanone	50.270	224	$C_{12}H_{16}O_4$	181	106370	0.23	MS	2648
**M145**	Propioveratrone	50.740	194	$C_{11}H_{14}O_3$	165	65475	0.14	MS	2686

No.	Compounds	t_R (min)	MW	Formula	Ion	Area	C (%)	Identification	RI
**M149**	1-Hydroxy-2-(prop-2-enyl)-4,5-methylenedioxybenzene	51.670	178	$C_{10}H_{10}O_3$	135	40318	0.09	MS	2750
**M150**	3-(2,4,6-Trimethoxyphenyl)-2-butanone	51.850	238	$C_{13}H_{18}O_4$	149	47506	0.10	MS	2761
**M152**	1-Hydroxy-2-(prop-2-enyl)-4,5-methylenedioxybenzene isomer	52.665	178	$C_{10}H_{10}O_3$	178	34823	0.07	MS	2814
**M153**	Syringic acid	55.055	198	$C_9H_{10}O_5$	198	26757	0.06	MS	2934

The note of Table S2 is also suitable for Table S3.

Table S4. The information of 24 VOCs identified in the blood of rats in ARR powder-treated group by HS-SPME-GC-MS

No.	Compounds	t_R (min)	MW	Formula	Ion	Area	C (%)	Identification	RI
**M20**	*dl*-Camphor	15.305	152	$C_{10}H_{16}O$	95	11894	0.11	MS, RI	1508
**M22**	Eucarvone	17.060	150	$C_{10}H_{14}O$	107	69543	0.65	MS, REF	1543
**M37**	Estragole	23.905	148	$C_{10}H_{12}O$	148	30309	0.28	MS, RI, REF	1664
**M44**	Borneol	25.875	154	$C_{10}H_{18}O$	95	13032	0.12	MS, RI	1695
**M60**	3,5-Dimethoxytoluene	34.260	152	$C_9H_{12}O_2$	152	2675747	25.02	MS, RI, REF	1841
**M64**	Safrole	35.110	162	$C_{10}H_{10}O_2$	162	2539402	23.74	MS, RI, REF	1863
**M74**	Thymoquinone	38.880	164	$C_{10}H_{12}O_2$	149	3089	0.03	MS, RI	1980
**M79**	Methyleugenol	39.715	178	$C_{11}H_{14}O_2$	178	501888	4.69	MS, RI, REF	2011
**M82**	2,3,5-Trimethoxytoluene	40.415	182	$C_{10}H_{14}O_3$	167	1913228	17.89	REF	2040
**M83**	3,4,5-Trimethoxytoluene	40.610	182	$C_{10}H_{14}O_3$	182	2107637	19.71	MS, RI, REF	2048
**M84**	3,4,5-Trimethoxybenzoic acid	40.780	212	$C_{10}H_{12}O_5$	212	13334	0.12	MS	2056
**M87**	*E*-Isocroweacin	41.755	192	$C_{11}H_{12}O_3$	192	172532	1.61	MS	2097
**M95**	4-Methoxysafrole	43.745	192	$C_{11}H_{12}O_3$	192	234811	2.20	MS, RI	2189
**M97**	2,4,5-Trimethoxybenzoic acid	43.815	212	$C_{10}H_{12}O_5$	212	14710	0.14	MS	2192
**M99**	*p*-Methoxypropiophenone	43.910	164	$C_{10}H_{12}O_2$	135	17908	0.17	MS	2197
**M104**	Piperonal	44.340	150	$C_8H_6O_3$	149	7544	0.07	MS, RI	2219
**M107**	Elemicin	44.505	208	$C_{12}H_{16}O_3$	208	19365	0.18	MS, RI, REF	2228
**M108**	3,4,5-Trimethoxytoluene isomer	44.765	182	$C_{10}H_{14}O_3$	182	10326	0.10	MS	2242
**M112**	β-Asarone	45.180	208	$C_{12}H_{16}O_3$	208	4593	0.04	MS, RI, REF	2264
**M116**	3,4-Methylenedioxyacetophenone	46.400	164	$C_9H_8O_3$	149	14711	0.14	MS	2337
**M122**	3,4-Methylenedioxypropiophenone	47.395	178	$C_{10}H_{10}O_3$	149	169092	1.58	MS, REF	2405
**M131**	1-(3,4-Methylenedioxyphenyl)-propane-1-ol	48.770	180	$C_{10}H_{12}O_3$	151	15574	0.15	MS	2520
**M138**	Kakuol	49.295	194	$C_{10}H_{10}O_4$	165	98931	0.92	MS, REF	2567
**M148**	2',4'-Dimethoxy-3'-methylpropiophenone	51.435	208	$C_{12}H_{16}O_3$	179	36560	0.34	MS	2734

The note of Table S2 is also suitable for Table S4.

Table S5. The information of 16 VOCs identified in the brain of rats in ARR powder-treated group by HS-SPME-GC-MS

No.	Compounds	t_R (min)	MW	Formula	Ion	Area	C (%)	Identification	RI
**M22**	Eucarvone	17.095	150	$C_{10}H_{14}O$	107	25300	0.36	MS, REF	1544
**M37**	Estragole	23.970	148	$C_{10}H_{12}O$	148	32455	0.47	MS, RI, REF	1664
**M43**	4-Ethylbenzaldehyde	25.830	134	$C_9H_{10}O$	134	169319	2.44	MS, RI	1693
**M60**	3,5-Dimethoxytoluene	34.280	152	$C_9H_{12}O_2$	152	2476313	35.66	MS, RI, REF	1841
**M64**	Safrole	35.140	162	$C_{10}H_{10}O_2$	162	1936097	27.88	MS, RI, REF	1863
**M79**	Methyleugenol	39.720	178	$C_{11}H_{14}O_2$	178	398322	5.74	MS, RI, REF	2011
**M82**	2,3,5-Trimethoxytoluene	40.420	182	$C_{10}H_{14}O_3$	167	750919	10.81	REF	2041
**M83**	3,4,5-Trimethoxytoluene	40.615	182	$C_{10}H_{14}O_3$	182	641840	9.24	MS, RI, REF	2049
**M87**	*E*-Isocroweacin	41.760	192	$C_{11}H_{12}O_3$	192	88522	1.27	MS	2096
**M95**	4-Methoxysafrole	43.755	192	$C_{11}H_{12}O_3$	192	214327	3.09	MS, RI	2190
**M99**	*p*-Methoxypropiophenone	43.915	164	$C_{10}H_{12}O_2$	135	10483	0.15	MS	2197
**M107**	Elemicin	44.500	208	$C_{12}H_{16}O_3$	208	11114	0.16	MS, RI, REF	2228
**M108**	3,4,5-Trimethoxytoluene isomer	44.770	182	$C_{10}H_{14}O_3$	182	3793	0.05	MS	2242
**M122**	3,4-Methylenedioxypropiophenone	47.400	178	$C_{10}H_{10}O_3$	149	100124	1.44	MS, REF	2406
**M138**	Kakuol	49.300	194	$C_{10}H_{10}O_4$	165	67174	0.97	MS, REF	2567
**M148**	2',4'-Dimethoxy-3'-methylpropiophenone	51.455	208	$C_{12}H_{16}O_3$	179	18747	0.27	MS	2736

The note of Table S2 is also suitable for Table S5.

Table S6. The information of 15 VOCs identified in the heart of rats in ARR powder-treated group by HS-SPME-GC-MS

No.	Compounds	t_R (min)	MW	Formula	Ion	Area	C (%)	Identification	RI
**M20**	*dl*-Camphor	15.305	152	$C_{10}H_{16}O$	95	5247	0.13	MS, RI	1508
**M22**	Eucarvone	17.060	150	$C_{10}H_{14}O$	107	17260	0.42	MS, REF	1544
**M37**	Estragole	23.905	148	$C_{10}H_{12}O$	148	14670	0.35	MS, RI, REF	1665
**M43**	4-Ethylbenzaldehyde	25.830	134	$C_9H_{10}O$	134	37670	0.91	MS, RI	1694
**M60**	3,5-Dimethoxytoluene	34.260	152	$C_9H_{12}O_2$	152	1447286	34.93	MS, RI, REF	1841
**M64**	Safrole	35.110	162	$C_{10}H_{10}O_2$	162	981957	23.70	MS, RI, REF	1863
**M79**	Methyleugenol	39.715	178	$C_{11}H_{14}O_2$	178	263514	6.36	MS, RI, REF	2011
**M82**	2,3,5-Trimethoxytoluene	40.415	182	$C_{10}H_{14}O_3$	167	544941	13.15	REF	2040
**M83**	3,4,5-Trimethoxytoluene	40.610	182	$C_{10}H_{14}O_3$	182	460060	11.10	MS, RI, REF	2049
**M87**	*E*-Isocroweacin	41.755	192	$C_{11}H_{12}O_3$	192	51956	1.25	MS	2096
**M95**	4-Methoxysafrole	43.745	192	$C_{11}H_{12}O_3$	192	139456	3.37	MS, RI	2189
**M107**	Elemicin	44.505	208	$C_{12}H_{16}O_3$	208	9808	0.24	MS, RI, REF	2228
**M122**	3,4-Methylenedioxypropiophenone	47.395	178	$C_{10}H_{10}O_3$	149	92509	2.23	MS, REF	2406
**M138**	Kakuol	49.295	194	$C_{10}H_{10}O_4$	165	60200	1.45	MS, REF	2567
**M148**	2',4'-Dimethoxy-3'-methylpropiophenone	51.435	208	$C_{12}H_{16}O_3$	179	17005	0.41	MS	2735

The note of Table S2 is also suitable for Table S6.

Table S7. The information of 15 VOCs identified in the lung of rats in ARR powder-treated group by HS-SPME-GC-MS

No.	Compounds	t_R (min)	MW	Formula	Ion	Area	C (%)	Identification	RI
**M20**	*dl*-Camphor	15.290	152	$C_{10}H_{16}O$	95	4213	0.12	MS, RI	1507
**M22**	Eucarvone	17.055	150	$C_{10}H_{14}O$	107	11913	0.33	MS, REF	1543
**M37**	Estragole	23.905	148	$C_{10}H_{12}O$	148	18888	0.53	MS, RI, REF	1663
**M60**	3,5-Dimethoxytoluene	34.255	152	$C_9H_{12}O_2$	152	1267567	35.43	MS, RI, REF	1840
**M64**	Safrole	35.110	162	$C_{10}H_{10}O_2$	162	979939	27.39	MS, RI, REF	1863
**M79**	Methyleugenol	39.720	178	$C_{11}H_{14}O_2$	178	242963	6.79	MS, RI, REF	2011
**M82**	2,3,5-Trimethoxytoluene	40.415	182	$C_{10}H_{14}O_3$	167	389394	10.89	REF	2040
**M83**	3,4,5-Trimethoxytoluene	40.610	182	$C_{10}H_{14}O_3$	182	327900	9.17	MS, RI, REF	2048
**M87**	*E*-Isocroweacin	41.745	192	$C_{11}H_{12}O_3$	192	31167	0.87	MS	2096
**M93**	Eugenol	43.255	164	$C_{10}H_{12}O_2$	164	53666	1.50	MS, RI, REF	2166
**M99**	*p*-Methoxypropiophenone	43.920	164	$C_{10}H_{12}O_2$	135	10120	0.28	MS	2197
**M115**	1,2,4-Trimethoxybenzene isomer	46.360	168	$C_9H_{12}O_3$	153	6382	0.18	MS	2334
**M122**	3,4-Methylenedioxypropiophenone	47.400	178	$C_{10}H_{10}O_3$	149	123130	3.44	MS, REF	2406
**M138**	Kakuol	49.300	194	$C_{10}H_{10}O_4$	165	85328	2.39	MS, REF	2567
**M148**	2',4'-Dimethoxy-3'-methylpropiophenone	51.445	208	$C_{12}H_{16}O_3$	179	24654	0.69	MS	2735

The note of Table S2 is also suitable for Table S7.

Table S8. The information of 17 VOCs identified in the spleen of rats in ARR powder-treated group by HS-SPME-GC-MS

No.	Compounds	t_R (min)	MW	Formula	Ion	Area	C (%)	Identification	RI
**M22**	Eucarvone	17.060	150	$C_{10}H_{14}O$	107	46777	0.71	MS, REF	1543
**M37**	Estragole	23.905	148	$C_{10}H_{12}O$	148	20289	0.31	MS, RI, REF	1664
**M44**	Borneol	25.875	154	$C_{10}H_{18}O$	95	34582	0.52	MS, RI	1694
**M60**	3,5-Dimethoxytoluene	34.260	152	$C_9H_{12}O_2$	152	1862216	28.13	MS, RI, REF	1840
**M64**	Safrole	35.110	162	$C_{10}H_{10}O_2$	162	1427886	21.57	MS, RI, REF	1863
**M79**	Methyleugenol	39.715	178	$C_{11}H_{14}O_2$	178	902632	13.63	MS, RI, REF	2011
**M82**	2,3,5-Trimethoxytoluene	40.415	182	$C_{10}H_{14}O_3$	167	972738	14.69	REF	2040
**M83**	3,4,5-Trimethoxytoluene	40.610	182	$C_{10}H_{14}O_3$	182	588416	8.89	MS, RI, REF	2048
**M84**	3,4,5-Trimethoxybenzoic acid	40.780	212	$C_{10}H_{12}O_5$	212	6023	0.09	MS	2056
**M87**	*E*-Isocroweacin	41.755	192	$C_{11}H_{12}O_3$	192	115040	1.74	MS	2096
**M93**	Eugenol	43.250	164	$C_{10}H_{12}O_2$	164	21769	0.33	MS, RI, REF	2166
**M95**	4-Methoxysafrole	43.745	192	$C_{11}H_{12}O_3$	192	281324	4.25	MS, RI	2189
**M99**	*p*-Methoxypropiophenone	43.910	164	$C_{10}H_{12}O_2$	135	14283	0.22	MS	2197
**M122**	3,4-Methylenedioxypropiophenone	47.395	178	$C_{10}H_{10}O_3$	149	115466	1.74	MS, REF	2405
**M131**	1-(3,4-Methylenedioxyphenyl)-propane-1-ol	48.770	180	$C_{10}H_{12}O_3$	151	7836	0.12	MS	2521
**M138**	Kakuol	49.295	194	$C_{10}H_{10}O_4$	165	162096	2.45	MS, REF	2567
**M148**	2',4'-Dimethoxy-3'-methylpropiophenone	51.435	208	$C_{12}H_{16}O_3$	179	40977	0.62	MS	2734

The note of Table S2 is also suitable for Table S8.

Table S9. The information of 28 VOCs identified in the liver of rats in ARR powder-treated group by HS-SPME-GC-MS

No.	Compounds	t_R (min)	MW	Formula	Ion	Area	C (%)	Identification	RI
**M9**	Eucalyptol	7.095	154	$C_{10}H_{18}O$	81	5195	0.03	MS, RI	1203
**M20**	*dl*-Camphor	15.305	152	$C_{10}H_{16}O$	95	58391	0.29	MS, RI	1507
**M22**	Eucarvone	17.060	150	$C_{10}H_{14}O$	107	566692	2.83	MS, REF	1543
**M37**	Estragole	23.905	148	$C_{10}H_{12}O$	148	98101	0.49	MS, RI, REF	1664
**M40**	Eucarvone isomer	24.990	150	$C_{10}H_{14}O$	107	14115	0.07	MS	1680
**M46**	Piperitone	26.610	152	$C_{10}H_{16}O$	82	12951	0.06	MS, RI	1706
**M49**	Berbenone	28.190	150	$C_{10}H_{14}O$	150	21376	0.11	MS, RI	1731
**M60**	3,5-Dimethoxytoluene	34.260	152	$C_9H_{12}O_2$	152	7085640	35.35	MS, RI, REF	1841
**M64**	Safrole	35.110	162	$C_{10}H_{10}O_2$	162	5028955	25.09	MS, RI, REF	1863
**M70**	3,4-Methylenedioxyanisole	37.450	152	$C_8H_8O_3$	152	3781	0.02	MS	1931
**M77**	2,4-Dimethylanisole	39.485	136	$C_9H_{12}O$	121	3128	0.02	MS	2002
**M79**	Methyleugenol	39.715	178	$C_{11}H_{14}O_2$	178	1288382	6.43	MS, RI, REF	2011
**M81**	Isosafrole	39.925	162	$C_{10}H_{10}O_2$	162	9390	0.05	MS, RI	2020
**M82**	2,3,5-Trimethoxytoluene	40.415	182	$C_{10}H_{14}O_3$	167	2262002	11.28	REF	2040
**M83**	3,4,5-Trimethoxytoluene	40.610	182	$C_{10}H_{14}O_3$	182	1894040	9.45	MS, RI, REF	2048
**M84**	3,4,5-Trimethoxybenzoic acid	40.780	212	$C_{10}H_{12}O_5$	212	9546	0.05	MS	2056
**M87**	*E*-Isocroweacin	41.755	192	$C_{11}H_{12}O_3$	192	261667	1.31	MS	2096
**M88**	Dihydroeugenol	41.970	166	$C_{10}H_{14}O_2$	137	13873	0.07	MS, RI	2106
**M93**	Eugenol	43.250	164	$C_{10}H_{12}O_2$	164	47780	0.24	MS, RI, REF	2166
**M95**	4-Methoxysafrole	43.745	192	$C_{11}H_{12}O_3$	192	762872	3.81	MS, RI	2189
**M97**	2,4,5-Trimethoxybenzoic acid	43.815	212	$C_{10}H_{12}O_5$	212	12635	0.06	MS	2193
**M107**	Elemicin	44.505	208	$C_{12}H_{16}O_3$	208	37358	0.19	MS, RI, REF	2228
**M108**	3,4,5-Trimethoxytoluene isomer	44.765	182	$C_{10}H_{14}O_3$	182	9830	0.05	MS	2242
**M112**	β-Asarone	45.180	208	$C_{12}H_{16}O_3$	208	5206	0.03	MS, RI, REF	2264
**M122**	3,4-Methylenedioxypropiophenone	47.395	178	$C_{10}H_{10}O_3$	149	181736	0.91	MS, REF	2405
**M131**	1-(3,4-Methylenedioxyphenyl)-propane-1-ol	48.770	180	$C_{10}H_{12}O_3$	151	24984	0.12	MS	2520
**M138**	Kakuol	49.295	194	$C_{10}H_{10}O_4$	165	51950	0.26	MS, REF	2567
**M148**	2',4'-Dimethoxy-3'-methylpropiophenone	51.435	208	$C_{12}H_{16}O_3$	179	11636	0.06	MS	2735

The note of Table S2 is also suitable for Table S9.

Table S10. The information of 42 VOCs identified in the kidney of rats in ARR powder-treated group by HS-SPME-GC-MS

No.	Compounds	t_R (min)	MW	Formula	Ion	Area	C (%)	Identification	RI
**M9**	Eucalyptol	7.095	154	$C_{10}H_{18}O$	81	12615	0.06	MS, RI	1205
**M15**	*cis*-Limonene oxide	13.135	152	$C_{10}H_{16}O$	137	28661	0.15	MS, RI	1441
**M20**	*dl*-Camphor	15.305	152	$C_{10}H_{16}O$	95	308053	1.58	MS, RI	1508
**M22**	Eucarvone	17.060	150	$C_{10}H_{14}O$	107	1868768	9.59	MS, REF	1544
**M28**	Fenchol	18.920	154	$C_{10}H_{18}O$	81	13206	0.07	MS, RI	1581
**M35**	Umbellulone	21.815	150	$C_{10}H_{14}O$	108	14764	0.08	MS, RI	1631
**M37**	Estragole	23.905	148	$C_{10}H_{12}O$	148	34485	0.18	MS, RI, REF	1664
**M39**	Verbenol	24.570	152	$C_{10}H_{16}O$	57	6167	0.03	MS, RI	1674
**M40**	Eucarvone isomer	24.990	150	$C_{10}H_{14}O$	107	47889	0.25	MS	1680
**M41**	Verbenone	25.240	150	$C_{10}H_{14}O$	107	14711	0.08	MS, RI	1684
**M44**	Borneol	25.875	154	$C_{10}H_{18}O$	95	736722	3.78	MS, RI	1695
**M46**	Piperitone	26.610	152	$C_{10}H_{16}O$	82	55510	0.28	MS, RI	1706
**M49**	Berbenone	28.190	150	$C_{10}H_{14}O$	150	78325	0.40	MS, RI	1731
**M60**	3,5-Dimethoxytoluene	34.260	152	$C_9H_{12}O_2$	152	4634721	23.78	MS, RI, REF	1841
**M62**	2,3-Dimethyldecahydronaphthalene	34.995	166	$C_{12}H_{22}$	95	13308	0.07	MS	1859
**M64**	Safrole	35.110	162	$C_{10}H_{10}O_2$	162	2475969	12.70	MS, RI, REF	1863
**M67**	Verbenone isomer	36.360	150	$C_{10}H_{14}O$	150	19107	0.10	MS	1895
**M74**	Thymoquinone	38.880	164	$C_{10}H_{12}O_2$	149	42886	0.22	MS	1981
**M79**	Methyleugenol	39.715	178	$C_{11}H_{14}O_2$	178	877462	4.50	MS, RI, REF	2011
**M81**	Isosafrole	39.925	162	$C_{10}H_{10}O_2$	104	1796	0.01	MS, RI	2020
**M82**	2,3,5-Trimethoxytoluene	40.415	182	$C_{10}H_{14}O_3$	167	2581811	13.25	REF	2041
**M83**	3,4,5-Trimethoxytoluene	40.610	182	$C_{10}H_{14}O_3$	182	2784807	14.29	MS, RI, REF	2048
**M84**	3,4,5-Trimethoxybenzoic acid	40.780	212	$C_{10}H_{12}O_5$	212	11601	0.06	MS	2055
**M87**	*E*-Isocroweacin	41.755	192	$C_{11}H_{12}O_3$	192	286084	1.47	MS	2096
**M88**	Dihydroeugenol	41.970	166	$C_{10}H_{14}O_2$	137	20178	0.10	MS, RI	2106
**M93**	Eugenol	43.250	164	$C_{10}H_{12}O_2$	164	220027	1.13	MS, RI, REF	2166
**M94**	1,2,4-Trimethoxybenzene isomer	43.565	168	$C_9H_{12}O_3$	153	336281	1.73	MS	2181
**M95**	4-Methoxysafrole	43.745	192	$C_{11}H_{12}O_3$	192	326765	1.68	MS, RI	2189
**M99**	*p*-Methoxypropiophenone	43.910	164	$C_{10}H_{12}O_2$	135	18242	0.09	MS	2197
**M107**	Elemicin	44.505	208	$C_{12}H_{16}O_3$	208	23812	0.12	MS, RI, REF	2228
**M108**	3,4,5-Trimethoxytoluene isomer	44.765	182	$C_{10}H_{14}O_3$	182	8457	0.04	MS	2242
**M112**	β-Asarone	45.180	208	$C_{12}H_{16}O_3$	208	4704	0.02	MS, RI, REF	2264
**M115**	1,2,4-Trimethoxybenzene isomer	46.360	168	$C_9H_{12}O_3$	153	63038	0.32	MS	2334
**M117**	Chavicol	46.605	134	$C_9H_{10}O$	134	4596	0.02	MS, RI	2351
**M122**	3,4-Methylenedioxypropiophenone	47.395	178	$C_{10}H_{10}O_3$	149	196067	1.01	MS, REF	2405
**M127**	Kakuol isomer	47.895	194	$C_{10}H_{10}O_4$	165	88408	0.45	MS	2447
**M131**	1-(3,4-Methylenedioxyphenyl)-propane-1-ol	48.770	180	$C_{10}H_{12}O_3$	151	10303	0.05	MS	2521
**M138**	Kakuol	49.295	194	$C_{10}H_{10}O_4$	165	1134627	5.82	MS, REF	2567
**M139**	2',4'-Dimethoxy-3'-methylpropiophenone isomer	49.690	208	$C_{12}H_{16}O_3$	179	16160	0.08	MS	2602
**M140**	Xanthoxylin	49.760	196	$C_{10}H_{10}O_4$	181	13113	0.07	MS	2608
**M147**	Dihydromethyleugenol	51.135	180	$C_{11}H_{16}O_2$	151	17373	0.09	MS	2714
**M148**	2',4'-Dimethoxy-3'-methylpropiophenone	51.435	208	$C_{12}H_{16}O_3$	179	38618	0.20	MS	2735

The note of Table S2 is also suitable for Table S10.

Table S11. The information of 62 VOCs identified in the stomach of rats in ARR powder-treated group by HS-SPME-GC-MS

No.	Compounds	t_R (min)	MW	Formula	Ion	Area	C (%)	Identification	RI
**M3**	β-Pinene	4.955	136	$C_{10}H_{16}$	93	17937	0.01	MS, RI, REF	1099
**M5**	3-Carene	5.700	136	$C_{10}H_{16}$	93	23745	0.01	MS, RI	1135
**M6**	β-Myrcene	6.080	136	$C_{10}H_{16}$	93	9704	0.00	MS, RI	1154
**M7**	*d*-4-Carene	6.405	136	$C_{10}H_{16}$	93	3437	0.00	MS, RI	1170
**M8**	Limonene	6.830	136	$C_{10}H_{16}$	93	10445	0.00	MS, RI, REF	1191
**M9**	Eucalyptol	7.095	154	$C_{10}H_{18}O$	81	10640	0.01	MS, RI	1203
**M10**	*o*-Cymene	8.630	134	$C_{10}H_{14}$	119	55805	0.03	MS, RI	1268
**M11**	Terpinolene	8.895	136	$C_{10}H_{16}$	93	5099	0.00	MS, RI	1280
**M14**	*p*-Cymenene	12.885	132	$C_{10}H_{12}$	117	20044	0.01	MS, RI	1433
**M20**	*dl*-Camphor	15.305	152	$C_{10}H_{16}O$	95	105363	0.05	MS, RI	1507
**M21**	1-Pentadecene	16.070	210	$C_{15}H_{30}$	69	59636	0.03	MS, RI	1523
**M22**	Eucarvone	17.060	150	$C_{10}H_{14}O$	107	984854	0.47	MS, REF	1543
**M23**	*l*-Aristolene	17.560	204	$C_{15}H_{24}$	105	21674	0.01	MS, RI	1553
**M26**	Bornyl acetate	18.305	196	$C_{12}H_{20}O_2$	95	7402	0.00	MS, RI	1568
**M27**	1(10)-Aristolene	18.760	204	$C_{15}H_{24}$	161	140429	0.07	MS, RI	1577
**M29**	Thymol methyl ether	19.390	164	$C_{11}H_{16}O$	149	156458	0.07	MS, RI	1590
**M30**	*l*-Aristolene isomer	19.650	204	$C_{15}H_{24}$	105	59094	0.03	MS	1596
**M31**	β-Cyclocitral	20.510	152	$C_{10}H_{16}O$	137	13979	0.01	MS, RI	1610
**M34**	δ-Guaiene	21.535	204	$C_{15}H_{24}$	93	43622	0.02	MS, RI	1626
**M37**	Estragole	23.905	148	$C_{10}H_{12}O$	148	790331	0.37	MS, RI, REF	1664
**M42**	α-Terpineol acetate	25.300	196	$C_{12}H_{20}O_2$	121	38472	0.02	MS, RI	1685
**M44**	Borneol	25.875	154	$C_{10}H_{18}O$	95	3043428	1.44	MS, RI	1694
**M46**	Piperitone	26.610	152	$C_{10}H_{16}O$	82	32199	0.02	MS, RI	1706
**M49**	Berbenone	28.190	150	$C_{10}H_{14}O$	150	16935	0.01	MS, RI	1730
**M56**	Cuparene	32.520	202	$C_{15}H_{22}$	81	15065	0.01	MS, RI	1797
**M59**	*cis*-Carveol	34.050	152	$C_{10}H_{16}O$	109	7714	0.00	MS, RI	1836
**M60**	3,5-Dimethoxytoluene	34.260	152	$C_9H_{12}O_2$	152	35161970	16.67	MS, RI, REF	1842
**M61**	*p*-Cymen-8-ol	34.660	150	$C_{10}H_{14}O$	135	101117	0.05	MS, RI	1852
**M64**	Safrole	35.110	162	$C_{10}H_{10}O_2$	162	46536941	22.07	MS, RI, REF	1865
**M70**	3,4-Methylenedioxyanisole	37.450	152	$C_8H_8O_3$	152	24961	0.01	MS	1931
**M73**	2-Allyl-1,4-dimethoxybenzene	38.535	178	$C_{11}H_{14}O_2$	178	38310	0.02	MS	1968
**M75**	Methyleugenol isomer	39.055	178	$C_{11}H_{14}O_2$	178	226534	0.11	MS	1986
**M79**	Methyleugenol	39.715	178	$C_{11}H_{14}O_2$	178	34994264	16.59	MS, RI, REF	2014
**M81**	Isosafrole	39.925	162	$C_{10}H_{10}O_2$	162	38093	0.02	MS, RI	2020
**M82**	2,3,5-Trimethoxytoluene	40.415	182	$C_{10}H_{14}O_3$	167	18837881	8.93	REF	2042
**M83**	3,4,5-Trimethoxytoluene	40.610	182	$C_{10}H_{14}O_3$	182	17072337	8.10	MS, RI, REF	2050
**M84**	3,4,5-Trimethoxybenzoic acid	40.780	212	$C_{10}H_{12}O_5$	212	186084	0.09	MS	2056
**M85**	Globulol	40.910	222	$C_{15}H_{26}O$	95	27361	0.01	MS, RI	2062
**M86**	1,2,4-Trimethoxybenzene	41.430	168	$C_9H_{12}O_3$	168	6800	0.00	MS, RI	2083
**M87**	*E*-Isocroweacin	41.755	192	$C_{11}H_{12}O_3$	192	9026023	4.28	MS	2097
**M89**	Spathulenol	42.110	220	$C_{15}H_{24}O$	205	18646	0.01	MS, RI	2113
**M91**	Viridiflorol	42.310	222	$C_{15}H_{26}O$	107	19235	0.01	MS, RI	2122
**M92**	Patchoulol	43.065	222	$C_{15}H_{26}O$	83	66947	0.03	MS, RI	2157
**M93**	Eugenol	43.250	164	$C_{10}H_{12}O_2$	164	198164	0.09	MS, RI, REF	2167
**M95**	4-Methoxysafrole	43.745	192	$C_{11}H_{12}O_3$	192	26607474	12.62	MS, RI	2191
**M101**	α-Bisabolol	44.200	222	$C_{15}H_{26}O$	69	46602	0.02	MS, RI	2212
**M105**	Isothymol	44.390	150	$C_{10}H_{14}O$	135	75209	0.04	MS, RI	2222
**M107**	Elemicin	44.505	208	$C_{12}H_{16}O_3$	208	1202961	0.57	MS, RI, REF	2228
**M108**	3,4,5-Trimethoxytoluene isomer	44.765	182	$C_{10}H_{14}O_3$	182	62708	0.03	MS	2242
**M110**	Isospathulenol	44.935	220	$C_{15}H_{24}O$	119	17807	0.01	MS, RI	2251
**M112**	β-Asarone	45.180	208	$C_{12}H_{16}O_3$	208	279835	0.13	MS, RI, REF	2264
**M119**	Kaurene	46.785	272	$C_{20}H_{32}$	257	9673	0.00	MS, RI	2363
**M122**	3,4-Methylenedioxypropiophenone	47.395	178	$C_{10}H_{10}O_3$	149	3304576	1.57	MS, REF	2406
**M123**	Apiol	47.510	222	$C_{12}H_{14}O_4$	222	15846	0.01	MS, RI	2415
**M127**	Kakuol isomer	47.895	194	$C_{10}H_{10}O_4$	165	274838	0.13	MS	2447
**M128**	Mellein	48.255	178	$C_{10}H_{10}O_3$	178	13794	0.01	MS, RI	2476
**M138**	Kakuol	49.295	194	$C_{10}H_{10}O_4$	165	8577238	4.07	MS, REF	2568
**M139**	2',4'-Dimethoxy-3'-methylpropiophenone isomer	49.690	208	$C_{12}H_{16}O_3$	179	126691	0.06	MS	2602
**M140**	Xanthoxylin	49.760	196	$C_{10}H_{10}O_4$	181	172365	0.08	MS	2608
**M144**	4-(Dimethoxymethyl)-1,2-dimethoxybenzene	50.615	212	$C_{11}H_{16}O_4$	181	22593	0.01	MS	2676
**M148**	2',4'-Dimethoxy-3'-methylpropiophenone	51.435	208	$C_{12}H_{16}O_3$	179	1763142	0.84	MS	2735
**M151**	1,2-Dimethoxy-4-(1,2-dimethoxyethyl)-benzene	52.195	226	$C_{12}H_{18}O_4$	181	26600	0.01	MS	2785

The note of Table S2 is also suitable for Table S11.

Table S12. The information of 34 VOCs identified in the small intestine of rats in ARR powder-treated group by HS-SPME-GC-MS

No.	Compounds	t_R (min)	MW	Formula	Ion	Area	C (%)	Identification	RI
**M5**	3-Carene	5.700	136	$C_{10}H_{16}$	93	5960	0.01	MS, RI	1136
**M9**	Eucalyptol	7.095	154	$C_{10}H_{18}O$	81	3970	0.01	MS, RI	1203
**M20**	*dl*-Camphor	15.305	152	$C_{10}H_{16}O$	95	38131	0.06	MS, RI	1507
**M22**	Eucarvone	17.060	150	$C_{10}H_{14}O$	107	342083	0.51	MS, REF	1543
**M29**	Thymol methyl ether	19.390	164	$C_{11}H_{16}O$	149	31430	0.05	MS, RI	1590
**M37**	Estragole	23.905	148	$C_{10}H_{12}O$	148	237350	0.35	MS, RI, REF	1664
**M44**	Borneol	25.875	154	$C_{10}H_{18}O$	95	965443	1.44	MS, RI	1694
**M60**	3,5-Dimethoxytoluene	34.260	152	$C_9H_{12}O_2$	152	13063274	19.44	MS, RI, REF	1841
**M64**	Safrole	35.110	162	$C_{10}H_{10}O_2$	162	16022242	23.84	MS, RI, REF	1864
**M75**	Methyleugenol isomer	39.055	178	$C_{11}H_{14}O_2$	178	47726	0.07	MS	1987
**M79**	Methyleugenol	39.715	178	$C_{11}H_{14}O_2$	178	12384980	18.43	MS, RI, REF	2012
**M81**	Isosafrole	39.925	162	$C_{10}H_{10}O_2$	162	8074	0.01	MS, RI	2020
**M82**	2,3,5-Trimethoxytoluene	40.415	182	$C_{10}H_{14}O_3$	167	6558575	9.76	REF	2041
**M83**	3,4,5-Trimethoxytoluene	40.610	182	$C_{10}H_{14}O_3$	182	5829924	8.68	MS, RI, REF	2049
**M84**	3,4,5-Trimethoxybenzoic acid	40.780	212	$C_{10}H_{12}O_5$	212	52814	0.08	MS	2056
**M85**	Globulol	40.910	222	$C_{15}H_{26}O$	95	4654	0.01	MS, RI	2061
**M87**	*E*-Isocroweacin	41.755	192	$C_{11}H_{12}O_3$	192	1938867	2.89	MS	2096
**M88**	Dihydroeugenol	41.970	166	$C_{10}H_{14}O_2$	137	19702	0.03	MS, RI	2106
**M89**	Spathulenol	42.110	220	$C_{15}H_{24}O$	205	3124	0.00	MS, RI	2113
**M91**	Viridiflorol	42.310	222	$C_{15}H_{26}O$	107	3628	0.01	MS, RI	2122
**M92**	Patchoulol	43.065	222	$C_{15}H_{26}O$	83	12351	0.02	MS, RI	2157
**M93**	Eugenol	43.250	164	$C_{10}H_{12}O_2$	164	59577	0.09	MS, RI, REF	2166
**M95**	4-Methoxysafrole	43.745	192	$C_{11}H_{12}O_3$	192	5769570	8.59	MS, RI	2190
**M99**	*p*-Methoxypropiophenone	43.910	164	$C_{10}H_{12}O_2$	135	106617	0.16	MS	2197
**M107**	Elemicin	44.505	208	$C_{12}H_{16}O_3$	208	294399	0.44	MS, RI, REF	2228
**M108**	3,4,5-Trimethoxytoluene isomer	44.765	182	$C_{10}H_{14}O_3$	182	14044	0.02	MS	2242
**M122**	3,4-Methylenedioxypropiophenone	47.395	178	$C_{10}H_{10}O_3$	149	786080	1.17	MS, REF	2405
**M127**	Kakuol isomer	47.895	194	$C_{10}H_{10}O_4$	165	69199	0.10	MS	2446
**M128**	Mellein	48.255	178	$C_{10}H_{10}O_3$	178	4164	0.01	MS, RI	2476
**M131**	1-(3,4-Methylenedioxyphenyl)-propane-1-ol	48.770	180	$C_{10}H_{12}O_3$	151	66510	0.10	MS	2520
**M138**	Kakuol	49.295	194	$C_{10}H_{10}O_4$	165	2012314	2.99	MS, REF	2567
**M139**	2',4'-Dimethoxy-3'-methylpropiophenone isomer	49.690	208	$C_{12}H_{16}O_3$	179	35644	0.05	MS	2602
**M140**	Xanthoxylin	49.760	196	$C_{10}H_{10}O_4$	181	37461	0.06	MS	2608
**M148**	2',4'-Dimethoxy-3'-methylpropiophenone	51.435	208	$C_{12}H_{16}O_3$	179	370511	0.55	MS	2735

The note of Table S2 is also suitable for Table S12.

Table S13. The information of 20 VOCs identified in the feces of rats in ARR decoction-treated group by HS-SPME-GC-MS

No.	Compounds	t_R (min)	MW	Formula	Ion	Area	C (%)	Identification	RI
**M12**	Tridecane	9.217	184	$C_{13}H_{28}$	184	3144	0.18	MS, RI, REF	1293
**M13**	Acetoin	9.328	88	$C_4H_8O_2$	88	108393	6.35	MS, RI	1298
**M20**	*dl*-Camphor	15.291	152	$C_{10}H_{16}O$	95	8076	0.47	MS, RI	1507
**M26**	Bornyl acetate	18.313	196	$C_{12}H_{20}O_2$	95	5905	0.35	MS, RI	1568
**M44**	Borneol	25.881	154	$C_{10}H_{18}O$	95	529068	31.00	MS, RI	1694
**M64**	Safrole	35.108	162	$C_{10}H_{10}O_2$	162	5797	0.34	MS, RI, REF	1863
**M79**	Methyleugenol	39.709	178	$C_{11}H_{14}O_2$	178	5220	0.31	MS, RI, REF	2011
**M81**	Isosafrole	39.931	162	$C_{10}H_{10}O_2$	162	20971	1.23	MS, RI	2020
**M82**	2,3,5-Trimethoxytoluene	40.407	182	$C_{10}H_{14}O_3$	167	5492	0.32	REF	2040
**M83**	3,4,5-Trimethoxytoluene	40.608	182	$C_{10}H_{14}O_3$	182	11413	0.67	MS, RI, REF	2049
**M93**	Eugenol	43.255	164	$C_{10}H_{12}O_2$	164	18756	1.10	MS, RI, REF	2167
**M95**	4-Methoxysafrole	43.747	192	$C_{11}H_{12}O_3$	192	11377	0.67	MS, RI	2190
**M98**	Thymol	43.870	150	$C_{10}H_{14}O$	135	110911	6.50	MS, RI	2197
**M102**	2-Aminoacetophenone	44.232	135	C_8H_9NO	135	51990	3.05	MS, RI	2214
**M105**	Isothymol	44.403	150	$C_{10}H_{14}O$	135	16787	0.98	MS, RI	2223
**M122**	3,4-Methylenedioxypropiophenone	47.392	178	$C_{10}H_{10}O_3$	149	31492	1.85	MS, REF	2406
**M127**	Kakuol isomer	47.901	194	$C_{10}H_{10}O_4$	165	28529	1.67	MS	2447
**M131**	1-(3,4-Methylenedioxyphenyl)-propane-1-ol	48.777	180	$C_{10}H_{12}O_3$	151	15360	0.90	MS	2521
**M138**	Kakuol	49.294	194	$C_{10}H_{10}O_4$	165	702552	41.16	MS, REF	2568
**M148**	2',4'-Dimethoxy-3'-methylpropiophenone	51.439	208	$C_{12}H_{16}O_3$	179	15469	0.91	MS	2735

The note of Table S2 is also suitable for Table S13.

Table S14. The information of 48 VOCs identified in the urine of rats in ARR decoction-treated group by HS-SPME-GC-MS

No.	Compounds	t_R (min)	MW	Formula	Ion	Area	C (%)	Identification	RI
**M20**	*dl*-Camphor	15.291	152	$C_{10}H_{16}O$	95	39912	0.14	MS, RI	1507
**M32**	Methyl benzoate	20.816	136	$C_8H_8O_2$	136	7703	0.03	MS, RI	1615
**M36**	*l*-Pinocarveol	23.114	152	$C_{10}H_{16}O$	92	39104	0.14	MS, RI	1651
**M44**	Borneol	25.881	154	$C_{10}H_{18}O$	95	1854171	6.67	MS, RI	1694
**M45**	Phellandral	26.439	154	$C_{10}H_{18}O$	109	51291	0.18	MS, RI	1703
**M53**	Myrtenol	31.916	152	$C_{10}H_{16}O$	108	4134	0.01	MS, RI	1788
**M55**	3,4-Dimethylbenzaldehyde	32.061	134	$C_9H_{10}O$	134	12324	0.04	MS, RI	1790
**M59**	*cis*-Carveol	34.055	152	$C_{10}H_{16}O$	109	17766	0.06	MS, RI	1835
**M63**	Guaiacol	35.000	124	$C_7H_8O_2$	124	140078	0.50	MS, RI	1860
**M64**	Safrole	35.108	162	$C_{10}H_{10}O_2$	162	3814	0.01	MS, RI, REF	1862
**M65**	Benzyl alcohol	35.662	108	C_7H_8O	108	132548	0.48	MS, RI	1877
**M68**	2-Phenylethanol	36.725	122	$C_8H_{10}O$	122	61343	0.22	MS, RI	1906
**M72**	Creosol	38.108	138	$C_8H_{10}O_2$	138	84630	0.30	MS, RI	1954
**M74**	Thymoquinone	38.885	164	$C_{10}H_{12}O_2$	149	47745	0.17	MS	1981
**M76**	6-Allyl-*o*-cresol	39.435	148	$C_{10}H_{12}O$	148	19770	0.07	MS	2000
**M79**	Methyleugenol	39.709	178	$C_{11}H_{14}O_2$	178	6433	0.02	MS, RI, REF	2011
**M82**	2,3,5-Trimethoxytoluene	40.407	182	$C_{10}H_{14}O_3$	167	30224	0.11	REF	2040
**M83**	3,4,5-Trimethoxytoluene	40.608	182	$C_{10}H_{14}O_3$	182	317898	1.14	MS, RI, REF	2048
**M88**	Dihydroeugenol	41.913	166	$C_{10}H_{14}O_2$	137	735442	2.64	MS, RI	2103
**M90**	Dihydroeugenol isomer	42.203	166	$C_{10}H_{14}O_2$	137	71840	0.26	MS	2117
**M93**	Eugenol	43.255	164	$C_{10}H_{12}O_2$	164	6582923	23.67	MS, RI, REF	2166
**M94**	1,2,4-Trimethoxybenzene isomer	43.589	168	$C_9H_{12}O_3$	153	635719	2.29	MS	2182
**M98**	Thymol	43.870	150	$C_{10}H_{14}O$	135	246441	0.89	MS, RI	2195
**M104**	Piperonal	44.354	150	$C_8H_6O_3$	149	329976	1.19	MS, RI	2220
**M109**	Methoxyeugenol isomer	44.889	194	$C_{11}H_{14}O_3$	194	16336	0.06	MS	2248
**M111**	*cis*-Isoeugenol	45.092	164	$C_{10}H_{12}O_2$	164	110098	0.40	MS, RI	2259
**M114**	1,2,3,4-Tetramethoxybenzene	45.605	198	$C_{10}H_{14}O_4$	198	27919	0.10	MS, RI	2286
**M115**	1,2,4-Trimethoxybenzene isomer	46.354	168	$C_9H_{12}O_3$	153	473718	1.70	MS	2334
**M116**	3,4-Methylenedioxyacetophenone	46.405	164	$C_9H_8O_3$	149	377751	1.36	MS	2337
**M117**	Chavicol	46.612	134	$C_9H_{10}O$	134	1405123	5.05	MS, RI	2351
**M118**	1,2,3-Trimethoxybenzene isomer	46.687	168	$C_9H_{12}O_3$	168	387185	1.39	MS	2356
**M121**	Methoxyeugenol isomer	47.304	194	$C_{11}H_{14}O_3$	194	10340	0.04	MS	2398
**M122**	3,4-Methylenedioxypropiophenone	47.392	178	$C_{10}H_{10}O_3$	149	99288	0.36	MS, REF	2405
**M126**	Methoxyeugenol isomer	47.845	194	$C_{11}H_{14}O_3$	194	227149	0.82	MS	2442
**M127**	Kakuol isomer	47.901	194	$C_{10}H_{10}O_4$	165	624505	2.25	MS	2447
**M128**	Mellein	48.262	178	$C_{10}H_{10}O_3$	178	29777	0.11	MS, RI	2476
**M130**	2,6-Dimethoxyacetophenone	48.585	180	$C_{10}H_{12}O_3$	180	5568	0.02	MS	2503
**M131**	1-(3,4-Methylenedioxyphenyl)-propane-1-ol	48.777	180	$C_{10}H_{12}O_3$	151	51400	0.18	MS	2521
**M132**	Piperonol	48.928	152	$C_8H_8O_3$	152	15637	0.06	MS	2534
**M134**	3-Methoxy-5-methylphenol	48.993	138	$C_8H_{10}O_2$	138	1316473	4.73	MS, RI	2540
**M137**	Methoxyeugenol	49.069	194	$C_{11}H_{14}O_3$	194	104000	0.37	MS, RI	2547
**M138**	Kakuol	49.294	194	$C_{10}H_{10}O_4$	165	10509763	37.79	MS, REF	2568
**M139**	2',4'-Dimethoxy-3'-methylpropiophenone isomer	49.690	208	$C_{12}H_{16}O_3$	179	19389	0.07	MS	2602
**M140**	Xanthoxylin	49.767	196	$C_{10}H_{10}O_4$	181	347256	1.25	MS	2608
**M143**	3,4,5-Trimethoxyphenyl-2-propanone	50.278	224	$C_{12}H_{16}O_4$	181	27492	0.10	MS	2649
**M146**	Propioveratrone	50.772	194	$C_{11}H_{14}O_3$	194	27897	0.10	MS	2687
**M148**	2',4'-Dimethoxy-3'-methylpropiophenone	51.439	208	$C_{12}H_{16}O_3$	179	69691	0.25	MS	2735
**M152**	1-Hydroxy-2-(prop-2-enyl)-4,5-methylenedioxybenzene	52.691	178	$C_{10}H_{10}O_3$	178	51784	0.19	MS	2815

The note of Table S2 is also suitable for Table S14.

Table S15. The information of 11 VOCs identified in the blood of rats in ARR decoction-treated group by HS-SPME-GC-MS

No.	Compounds	t_R (min)	MW	Formula	Ion	Area	C (%)	Identification	RI
**M74**	Thymoquinone	38.885	164	$C_{10}H_{12}O_2$	149	6902	1.48	MS	1981
**M82**	2,3,5-Trimethoxytoluene	40.407	182	$C_{10}H_{14}O_3$	167	32355	6.94	REF	2040
**M83**	3,4,5-Trimethoxytoluene	40.608	182	$C_{10}H_{14}O_3$	182	305666	65.52	MS, RI, REF	2048
**M97**	2,4,5-Trimethoxybenzoic acid	43.823	212	$C_{10}H_{12}O_5$	212	5925	1.27	MS	2193
**M104**	Piperonal	44.354	150	$C_8H_6O_3$	149	3635	0.78	MS, RI	2220
**M107**	Elemicin	44.508	208	$C_{12}H_{16}O_3$	208	2416	0.52	MS, RI, REF	2228
**M108**	3,4,5-Trimethoxytoluene isomer	44.767	182	$C_{10}H_{14}O_3$	182	10389	2.23	MS	2242
**M116**	3,4-Methylenedioxyacetophenone	46.405	164	$C_9H_8O_3$	149	25361	5.44	MS	2337
**M122**	3,4-Methylenedioxypropiophenone	47.392	178	$C_{10}H_{10}O_3$	149	14147	3.03	MS, REF	2405
**M138**	Kakuol	49.294	194	$C_{10}H_{10}O_4$	165	15402	3.30	MS, REF	2568
**M148**	2',4'-Dimethoxy-3'-methylpropiophenone	51.439	208	$C_{12}H_{16}O_3$	179	44317	9.50	MS	2735

The note of Table S2 is also suitable for Table S15.

Table S16. The information of five VOCs identified in the brain of rats in ARR decoction-treated group by HS-SPME-GC-MS

No.	Compounds	t_R (min)	MW	Formula	Ion	Area	C (%)	Identification	RI
**M82**	2,3,5-Trimethoxytoluene	40.407	182	$C_{10}H_{14}O_3$	167	7317	9.26	REF	2040
**M83**	3,4,5-Trimethoxytoluene	40.608	182	$C_{10}H_{14}O_3$	182	61790	78.18	MS, RI, REF	2048
**M107**	Elemicin	44.508	208	$C_{12}H_{16}O_3$	208	1032	1.31	MS, RI, REF	2228
**M122**	3,4-Methylenedioxypropiophenone	47.392	178	$C_{10}H_{10}O_3$	149	3709	4.69	MS, REF	2405
**M138**	Kakuol	49.294	194	$C_{10}H_{10}O_4$	165	5186	6.56	MS, REF	2567

The note of Table S2 is also suitable for Table S16.

Table S17. The information of five VOCs identified in the heart of rats in ARR decoction-treated group by HS-SPME-GC-MS

No.	Compounds	t_R (min)	MW	Formula	Ion	Area	C (%)	Identification	RI
**M82**	2,3,5-Trimethoxytoluene	40.407	182	$C_{10}H_{14}O_3$	167	6880	10.06	REF	2040
**M83**	3,4,5-Trimethoxytoluene	40.608	182	$C_{10}H_{14}O_3$	182	48886	71.51	MS, RI, REF	2048
**M93**	Eugenol	43.255	164	$C_{10}H_{12}O_2$	164	4184	6.12	MS, RI, REF	2167
**M122**	3,4-Methylenedioxypropiophenone	47.392	178	$C_{10}H_{10}O_3$	149	3580	5.24	MS, REF	2406
**M138**	Kakuol	49.294	194	$C_{10}H_{10}O_4$	165	4831	7.07	MS, REF	2568

The note of Table S2 is also suitable for Table S17.

Table S18. The information of seven VOCs identified in the lung of rats in ARR decoction-treated group by HS-SPME-GC-MS

No.	Compounds	t_R (min)	MW	Formula	Ion	Area	C (%)	Identification	RI
**M82**	2,3,5-Trimethoxytoluene	40.407	182	$C_{10}H_{14}O_3$	167	8005	7.86	REF	2040
**M83**	3,4,5-Trimethoxytoluene	40.608	182	$C_{10}H_{14}O_3$	182	52785	51.81	MS, RI, REF	2048
**M93**	Eugenol	43.255	164	$C_{10}H_{12}O_2$	164	10359	10.17	MS, RI, REF	2166
**M115**	1,2,4-Trimethoxybenzene isomer	46.354	168	$C_9H_{12}O_3$	153	1076	1.06	MS	2334
**M122**	3,4-Methylenedioxypropiophenone	47.392	178	$C_{10}H_{10}O_3$	149	2694	2.64	MS, REF	2405
**M138**	Kakuol	49.294	194	$C_{10}H_{10}O_4$	165	7861	7.72	MS, REF	2568
**M148**	2',4'-Dimethoxy-3'-methylpropiophenone	51.439	208	$C_{12}H_{16}O_3$	179	19097	18.75	MS	2735

The note of Table S2 is also suitable for Table S18.

Table S19. The information of nine VOCs identified in the spleen of rats in ARR decoction-treated group by HS-SPME-GC-MS

No.	Compounds	t_R (min)	MW	Formula	Ion	Area	C (%)	Identification	RI
**M64**	Safrole	35.108	162	$C_{10}H_{10}O_2$	162	416	0.39	MS, RI, REF	1862
**M79**	Methyleugenol	39.709	178	$C_{11}H_{14}O_2$	178	1640	1.53	MS, RI, REF	2011
**M82**	2,3,5-Trimethoxytoluene	40.407	182	$C_{10}H_{14}O_3$	167	9348	8.72	REF	2040
**M83**	3,4,5-Trimethoxytoluene	40.608	182	$C_{10}H_{14}O_3$	182	64942	60.58	MS, RI, REF	2048
**M93**	Eugenol	43.255	164	$C_{10}H_{12}O_2$	164	7274	6.79	MS, RI, REF	2166
**M107**	Elemicin	44.508	208	$C_{12}H_{16}O_3$	208	1458	1.36	MS, RI, REF	2228
**M122**	3,4-Methylenedioxypropiophenone	47.392	178	$C_{10}H_{10}O_3$	149	4218	3.93	MS, REF	2405
**M138**	Kakuol	49.294	194	$C_{10}H_{10}O_4$	165	7466	6.96	MS, REF	2568
**M148**	2',4'-Dimethoxy-3'-methylpropiophenone	51.439	208	$C_{12}H_{16}O_3$	179	10432	9.73	MS	2735

The note of Table S2 is also suitable for Table S19.

Table S20. The information of 18 VOCs identified in the liver of rats in ARR decoction-treated group by HS-SPME-GC-MS

No.	Compounds	t_R (min)	MW	Formula	Ion	Area	C (%)	Identification	RI
**M20**	*dl*-Camphor	15.291	152	$C_{10}H_{16}O$	95	2153	0.43	MS, RI	1507
**M43**	4-Ethylbenzaldehyde	25.836	134	$C_9H_{10}O$	134	56455	11.40	MS, RI	1694
**M60**	3,5-Dimethoxytoluene	34.265	152	$C_9H_{12}O_2$	152	1688	0.34	MS, RI, REF	1841
**M64**	Safrole	35.108	162	$C_{10}H_{10}O_2$	162	1238	0.25	MS, RI, REF	1862
**M74**	Thymoquinone	38.885	164	$C_{10}H_{12}O_2$	149	11724	2.37	MS	1981
**M79**	Methyleugenol	39.709	178	$C_{11}H_{14}O_2$	178	3244	0.65	MS, RI, REF	2011
**M82**	2,3,5-Trimethoxytoluene	40.407	182	$C_{10}H_{14}O_3$	167	30844	6.23	REF	2040
**M83**	3,4,5-Trimethoxytoluene	40.608	182	$C_{10}H_{14}O_3$	182	241327	48.72	MS, RI, REF	2048
**M88**	Dihydroeugenol	41.913	166	$C_{10}H_{14}O_2$	137	1906	0.38	MS, RI	2103
**M93**	Eugenol	43.255	164	$C_{10}H_{12}O_2$	164	18209	3.68	MS, RI	2166
**M107**	Elemicin	44.508	208	$C_{12}H_{16}O_3$	208	3721	0.75	MS, RI, REF	2228
**M108**	3,4,5-Trimethoxytoluene isomer	44.767	182	$C_{10}H_{14}O_3$	182	7437	1.50	MS	2242
**M115**	1,2,4-Trimethoxybenzene isomer	46.354	168	$C_9H_{12}O_3$	153	1694	0.34	MS	2334
**M117**	Chavicol	46.612	134	$C_9H_{10}O$	134	4782	0.97	MS, RI	2351
**M122**	3,4-Methylenedioxypropiophenone	47.392	178	$C_{10}H_{10}O_3$	149	18247	3.68	MS, REF	2405
**M131**	1-(3,4-Methylenedioxyphenyl)-propane-1-ol	48.777	180	$C_{10}H_{12}O_3$	151	2935	0.59	MS	2521
**M138**	Kakuol	49.294	194	$C_{10}H_{10}O_4$	165	42059	8.49	MS, REF	2568
**M148**	2',4'-Dimethoxy-3'-methylpropiophenone	51.439	208	$C_{12}H_{16}O_3$	179	45693	9.22	MS	2735

The note of Table S2 is also suitable for Table S20.

Table S21. The information of 26 VOCs identified in the kidney of rats in ARR decoction-treated group by HS-SPME-GC-MS

No.	Compounds	t_R (min)	MW	Formula	Ion	Area	C (%)	Identification	RI
**M15**	*cis*-Limonene oxide	13.131	152	$C_{10}H_{16}O$	137	3031	0.18	MS, RI	1441
**M20**	*dl*-Camphor	15.291	152	$C_{10}H_{16}O$	95	31696	1.93	MS, RI	1507
**M22**	Eucarvone	17.067	150	$C_{10}H_{14}O$	107	3448	0.21	MS, REF	1543
**M40**	Eucarvone isomer	25.014	150	$C_{10}H_{14}O$	107	3969	0.24	MS	1680
**M44**	Borneol	25.881	154	$C_{10}H_{18}O$	95	72403	4.42	MS, RI	1694
**M60**	3,5-Dimethoxytoluene	34.265	152	$C_9H_{12}O_2$	152	1907	0.12	MS, RI	1841
**M64**	Safrole	35.108	162	$C_{10}H_{10}O_2$	162	1811	0.11	MS, RI, REF	1862
**M66**	Verbenone isomer	36.215	150	$C_{10}H_{14}O$	150	9297	0.57	MS	1891
**M74**	Thymoquinone	38.885	164	$C_{10}H_{12}O_2$	149	139229	8.50	MS	1981
**M79**	Methyleugenol	39.709	178	$C_{11}H_{14}O_2$	178	5070	0.31	MS, RI	2011
**M82**	2,3,5-Trimethoxytoluene	40.407	182	$C_{10}H_{14}O_3$	167	55625	3.39	REF	2040
**M83**	3,4,5-Trimethoxytoluene	40.608	182	$C_{10}H_{14}O_3$	182	557810	34.04	MS, RI, REF	2048
**M84**	3,4,5-Trimethoxybenzoic acid	40.787	212	$C_{10}H_{12}O_5$	212	843	0.05	MS	2056
**M93**	Eugenol	43.255	164	$C_{10}H_{12}O_2$	164	232132	14.16	MS, RI	2166
**M97**	2,4,5-Trimethoxybenzoic acid	43.823	212	$C_{10}H_{12}O_5$	212	7656	0.47	MS	2193
**M107**	Elemicin	44.508	208	$C_{12}H_{16}O_3$	208	4490	0.27	MS, RI, REF	2228
**M108**	3,4,5-Trimethoxytoluene isomer	44.767	182	$C_{10}H_{14}O_3$	182	12478	0.76	MS	2242
**M115**	1,2,4-Trimethoxybenzene isomer	46.354	168	$C_9H_{12}O_3$	153	31550	1.93	MS	2334
**M117**	Chavicol	46.612	134	$C_9H_{10}O$	134	19571	1.19	MS, RI	2351
**M122**	3,4-Methylenedioxypropiophenone	47.392	178	$C_{10}H_{10}O_3$	149	19096	1.17	MS, REF	2405
**M127**	Kakuol isomer	47.901	194	$C_{10}H_{10}O_4$	165	8616	0.53	MS	2447
**M131**	1-(3,4-Methylenedioxyphenyl)-propane-1-ol	48.777	180	$C_{10}H_{12}O_3$	151	2958	0.18	MS	2521
**M138**	Kakuol	49.294	194	$C_{10}H_{10}O_4$	165	313796	19.15	MS, REF	2568
**M139**	2',4'-Dimethoxy-3'-methylpropiophenone isomer	49.690	208	$C_{12}H_{16}O_3$	179	41301	2.52	MS	2602
**M142**	2',4'-Dimethoxy-3'-methylpropiophenone isomer	50.226	208	$C_{12}H_{16}O_3$	179	8877	0.54	MS	2645
**M148**	2',4'-Dimethoxy-3'-methylpropiophenone	51.439	208	$C_{12}H_{16}O_3$	179	50176	3.06	MS	2735

The note of Table S2 is also suitable for Table S21.

Table S22. The information of 32 VOCs identified in the stomach of rats in ARR decoction-treated group by HS-SPME-GC-MS

No.	Compounds	t_R (min)	MW	Formula	Ion	Area	C (%)	Identification	RI
**M10**	*o*-Cymene	8.646	134	$C_{10}H_{14}$	119	2769	0.03	MS, RI	1269
**M19**	Pentadecane	14.953	212	$C_{15}H_{32}$	85	93104	1.17	MS, RI	1500
**M20**	*dl*-Camphor	15.291	152	$C_{10}H_{16}O$	95	1544	0.02	MS, RI	1507
**M44**	Borneol	25.881	154	$C_{10}H_{18}O$	95	18466	0.23	MS, RI	1694
**M60**	3,5-Dimethoxytoluene	34.265	152	$C_9H_{12}O_2$	152	2529	0.03	MS, RI, REF	1841
**M74**	Thymoquinone	38.885	164	$C_{10}H_{12}O_2$	149	4928	0.06	MS	1981
**M79**	Methyleugenol	39.709	178	$C_{11}H_{14}O_2$	178	80069	1.00	MS, RI, REF	2011
**M82**	2,3,5-Trimethoxytoluene	40.407	182	$C_{10}H_{14}O_3$	167	292668	3.66	REF	2040
**M83**	3,4,5-Trimethoxytoluene	40.608	182	$C_{10}H_{14}O_3$	182	1895207	23.72	MS, RI, REF	2048
**M84**	3,4,5-Trimethoxybenzoic acid	40.787	212	$C_{10}H_{12}O_5$	212	9511	0.12	MS	2056
**M86**	1,2,4-Trimethoxybenzene	41.437	168	$C_9H_{12}O_3$	168	3192	0.04	MS, RI	2083
**M93**	Eugenol	43.255	164	$C_{10}H_{12}O_2$	164	47827	0.60	MS, RI, REF	2166
**M95**	4-Methoxysafrole	43.747	192	$C_{11}H_{12}O_3$	192	7727	0.10	MS, RI	2189
**M97**	2,4,5-Trimethoxybenzoic acid	43.823	212	$C_{10}H_{12}O_5$	212	38856	0.49	MS	2193
**M107**	Elemicin	44.508	208	$C_{12}H_{16}O_3$	208	110865	1.39	MS, RI, REF	2228
**M108**	3,4,5-Trimethoxytoluene isomer	44.767	182	$C_{10}H_{14}O_3$	182	36703	0.46	MS	2242
**M112**	β-Asarone	45.186	208	$C_{12}H_{16}O_3$	208	16725	0.21	MS, RI, REF	2264
**M116**	3,4-Methylenedioxyacetophenone	46.405	164	$C_9H_8O_3$	149	9412	0.12	MS	2337
**M117**	Chavicol	46.612	134	$C_9H_{10}O$	134	12360	0.15	MS, RI	2351
**M122**	3,4-Methylenedioxypropiophenone	47.392	178	$C_{10}H_{10}O_3$	149	1239697	15.51	MS, REF	2405
**M126**	Methoxyeugenol isomer	47.845	194	$C_{11}H_{14}O_3$	194	8106	0.10	MS	2442
**M127**	Kakuol isomer	47.901	194	$C_{10}H_{10}O_4$	165	33782	0.42	MS	2447
**M128**	Mellein	48.262	178	$C_{10}H_{10}O_3$	178	6404	0.08	MS, RI	2476
**M131**	1-(3,4-Methylenedioxyphenyl)-propane-1-ol	48.777	180	$C_{10}H_{12}O_3$	151	54614	0.68	MS	2521
**M136**	4,6-Dimethoxy-phthalide	49.065	194	$C_{10}H_{10}O_4$	194	18922	0.24	MS	2546
**M138**	Kakuol	49.294	194	$C_{10}H_{10}O_4$	165	2362595	29.56	MS, RI, REF	2568
**M139**	2',4'-Dimethoxy-3'-methylpropiophenone isomer	49.690	208	$C_{12}H_{16}O_3$	179	107242	1.34	MS	2602
**M140**	Xanthoxylin	49.767	196	$C_{10}H_{10}O_4$	181	45209	0.57	MS	2608
**M144**	4-(Dimethoxymethyl)-1,2-dimethoxybenzene	50.621	212	$C_{11}H_{16}O_4$	181	15560	0.19	MS	2676
**M145**	2',4'-Dimethoxypropiophenone	50.760	194	$C_{11}H_{14}O_3$	194	5452	0.07	MS	2687
**M148**	2',4'-Dimethoxy-3'-methylpropiophenone	51.439	208	$C_{12}H_{16}O_3$	179	1392210	17.42	MS	2735
**M151**	1,2-Dimethoxy-4-(1,2-dimethoxyethyl)-benzene	52.205	226	$C_{12}H_{18}O_4$	181	17021	0.21	MS	2786

The note of Table S2 is also suitable for Table S22.

Table S23. The information of 19 VOCs identified in the small intestine of rats in ARR decoction-treated group by HS-SPME-GC-MS

No.	Compounds	t_R (min)	MW	Formula	Ion	Area	C (%)	Identification	RI
**M20**	*dl*-Camphor	15.291	152	$C_{10}H_{16}O$	95	2376	0.31	MS, RI	1507
**M60**	3,5-Dimethoxytoluene	34.265	152	$C_9H_{12}O_2$	152	2141	0.28	MS, RI, REF	1841
**M64**	Safrole	35.108	162	$C_{10}H_{10}O_2$	162	2499	0.32	MS, RI, REF	1863
**M79**	Methyleugenol	39.709	178	$C_{11}H_{14}O_2$	178	6909	0.90	MS, RI, REF	2011
**M82**	2,3,5-Trimethoxytoluene	40.407	182	$C_{10}H_{14}O_3$	167	26107	3.39	REF	2040
**M83**	3,4,5-Trimethoxytoluene	40.608	182	$C_{10}H_{14}O_3$	182	133284	17.30	MS, RI, REF	2049
**M93**	Eugenol	43.255	164	$C_{10}H_{12}O_2$	164	118092	15.33	MS, RI, REF	2167
**M98**	Thymol	43.870	150	$C_{10}H_{14}O$	135	4450	0.58	MS, RI	2197
**M107**	Elemicin	44.508	208	$C_{12}H_{16}O_3$	208	6991	0.91	MS, RI, REF	2228
**M108**	3,4,5-Trimethoxytoluene isomer	44.767	182	$C_{10}H_{14}O_3$	182	3286	0.43	MS	2242
**M115**	1,2,4-Trimethoxybenzene isomer	46.354	168	$C_9H_{12}O_3$	153	17542	2.28	MS	2335
**M117**	Chavicol	46.612	134	$C_9H_{10}O$	134	33855	4.39	MS, RI	2351
**M122**	3,4-Methylenedioxypropiophenone	47.392	178	$C_{10}H_{10}O_3$	149	40703	5.28	MS, REF	2406
**M131**	1-(3,4-Methylenedioxyphenyl)-propane-1-ol	48.777	180	$C_{10}H_{12}O_3$	151	9822	1.27	MS	2521
**M134**	3-Methoxy-5-methylphenol	48.993	138	$C_8H_{10}O_2$	138	74624	9.68	MS, RI	2540
**M138**	Kakuol	49.294	194	$C_{10}H_{10}O_4$	165	219150	28.44	MS, REF	2568
**M139**	2',4'-Dimethoxy-3'-methylpropiophenone isomer	49.690	208	$C_{12}H_{16}O_3$	179	5841	0.76	MS	2603
**M140**	Xanthoxylin	49.767	196	$C_{10}H_{10}O_4$	181	5654	0.73	MS	2608
**M148**	2',4'-Dimethoxy-3'-methylpropiophenone	51.439	208	$C_{12}H_{16}O_3$	179	57247	7.43	MS	2735

The note of Table S2 is also suitable for Table S23.
